# RebL1 is required for macronuclear structure stability and gametogenesis in *Tetrahymena thermophila*

**DOI:** 10.1007/s42995-024-00219-z

**Published:** 2024-03-26

**Authors:** Huijuan Hao, Yinjie Lian, Chenhui Ren, Sitong Yang, Min Zhao, Tao Bo, Jing Xu, Wei Wang

**Affiliations:** 1 Key Laboratory of Chemical Biology and Molecular Engineering of Ministry of Education, Institute of Biotechnology, Shanxi University, Taiyuan, 030006 China; 2 School of Life Science, Shanxi University, Taiyuan, 030006 China; 3 Shanxi Key Laboratory of Biotechnology, Taiyuan, 030006 China

**Keywords:** Gametogenesis, Histone chaperone, Macronuclear structure, RebL1, *Tetrahymena thermophila*

## Abstract

Histone modification and nucleosome assembly play important roles in chromatin-related processes. Histone chaperones form different complexes and coordinate histone transportation and assembly. Various histone chaperone complexes have been identified in different organisms. The ciliate protozoa (ciliates) have various chromatin structures and different nuclear morphology. However, histone chaperone components and functions of different subunits remain unclear in ciliates. *Tetrahymema thermophila* contains a transcriptionally active macronucleus (MAC) and a transcriptionally inactive micronucleus (MIC) which exhibit multiple replication and various chromatin remodeling progresses during vegetative growth and sexual developmental stages. Here, we found histone chaperone RebL1 not only localized evenly in the transcriptionally active MAC but also dynamically changed in the MIC during vegetative growth and sexual developmental stages. *REBL1* knockdown inhibited cellular proliferation. The macronuclear morphology became bigger in growing mutants. The abnormal macronuclear structure also occurred in the starvation stage. Furthermore, micronuclear meiosis was disturbed during sexual development, leading to a failure to generate new gametic nuclei. RebL1 potentially interacted with various factors involved in histone-modifying complexes and chromatin remodeling complexes in different developmental stages. *REBL1* knockdown affected expression levels of the genes involved in chromatin organization and transcription. Taken together, RebL1 plays a vital role in maintaining macronuclear structure stability and gametogenesis in *T. thermophila*.

**Supplementary Information:**

The online version contains supplementary material available at 10.1007/s42995-024-00219-z.

## Introduction

The organization of DNA into chromatin is critical for maintaining the integrity of the genome, facilitating proper gene expression control, and ensuring the accurate transmission of genetic information. The fundamental repeating unit of chromatin is the nucleosome, consisting of approximately 147 bp DNA wrapped in histone octamers assembled from histone H3-H4 tetramers and two histone H2A–H2B dimers. Tails of histones undergo different post-translational modifications that play important functions in chromatin remodeling and accessibility of DNA (Krebs 2007; Lee et al. 2010). Acetylation of histones neutralizes the positive charge of lysine, attenuates histone-DNA interactions, and opens the chromatin structures to promote transcription (Kuo et al. 1998; Shahbazian and Grunstein 2007). Histone acetylation modification is erased by histone deacetylases (HDACs), leading to the repression of transcription (Nakajima 2007; Wang et al. 2009). Heterochromatin is signified by repressive histone modifications, deacetylation, and methylation of histones H3K9 and H3K27. Different histone chaperones facilitate the orderly assembly of nucleosome structure and escort histone transport. Anti-silencing factor1 (ASF1) transfers H3–H4 heterodimers to chromatin assembly factor 1 (CAF-1) or histone regulation A (HIRA) for nucleosome assembly and contributes to heterochromatin formation (Feng et al. 2022; Yamane et al. 2011). CAF-1 is a highly conserved heterotrimeric complex, which consists of the p150, p60, RBBP4 [retinoblastoma binding protein 4, also called retinoblastoma-associated protein 48 (RbAp48)] in mammalian cells. RBBP4 has a histone H4 binding domain and seven WD40 repeats which form a seven-bladed β-propeller that promotes protein interaction (Kaushik et al. 2020). Vertebrates have two RBBP4 homologs, RBBP4 and RBBP7 (retinoblastoma binding protein 7), but only one orthologous gene in *Caenorhabditis elegans* (Lin53), *Drosophila melanogaster* (p55), and *Saccharomyces cerevisiae* (cac3) (Müthel et al. 2019; Nabeel-Shah et al. 2021; Wen et al. 2012). RBBP4 and RBBP7 form different chromatin modification complexes and chromatin remodeling complexes, including nucleosome remodeling histone deacetylase complex (NuRD) (Banach-Orlowska et al. 2009; Marhold et al. 2004), nucleosome remodeling factor (NURF) (Nowak et al. 2011), Sin3-Rpd3 complex (Vermaak et al. 1999; Zhao et al. 2020), polycomb repressive complex 2 (PRC2) (Grau et al. 2021; Wang et al. 2022), HAT1 complex (Ge et al. 2011), and CAF-1 complex (Cheloufi and Hochedlinger 2017; Hoek and Stillman 2003). The absence of mouse RBBP4 causes severe DNA damage, histone hyperacetylation, inner cell mass defects, and preimplantation lethality of embryos (Miao et al. 2020). RBBP4 physically interacts with histone deacetylase HDAC3 and favors the deacetylation of histones in mouse embryonic fibroblasts (Nicolas 2001). In glioblastoma, RBBP4 knockdown suppresses the expression of DNA methyltransferase and DNA recombinase RAD51 (Kitange et al. 2016; Nabeel-Shah et al. 2021). In chicken DT40 B cells, long-term exhaustion of RBBP4 results in replication abnormalities in S phase along with poor chromatin assembly (Satrimafitrah et al. 2016). In addition, heterochromatin protein 1 dissociates from periplasmic heterochromatin, and the acetylation level of H3K9 increases (Satrimafitrah et al. 2016). In *Drosophila*, the removal of p55 affects the expression level of E2F-regulated genes (Taylor-Harding et al. 2004). In yeast, Cac3/RBBP4 deletion decreases the silencing of telomeric genes and increases their lethality to ultraviolet radiation (Game and Kaufman 1999). In CD4^+^ T cell line, RBBP4 knockdown promotes HIV infection and virus particle generation (Wang et al. 2016).

Ciliated protozoa (ciliates) have various chromatin structures and nuclear morphologies. *Tetrahymena thermophila* has nuclear dimorphism. The somatic macronucleus (MAC) is polyploid and transcriptionally active, while the germline micronucleus (MIC) is diploid and transcriptionally silent during vegetative growth (Orias et al. 2011). During growth, the MIC divides mitotically while the MAC divides amitotically (Orias et al. 2011). The MIC begins to replicate during the late anaphase of division when it is separated from the MAC and positioned near the surface of the MAC in the G2 phase (Cole and Sugai 2012; Woodard et al. 1972). During sexual reproduction, one of the meiotic products is selected and initiates mitosis to produce gametic nuclei. The zygotic nuclei form by the exchange and fusion of gametic nuclei. The zygotic nucleus performs two rounds of mitosis and the parental MACs degrade gradually. Finally, the paired cells form exconjugants, each with two MACs and one MIC. The exconjugant restarts proliferation in a nutrient-sufficient environment (Cole and Sugai 2012). The MAC and MIC contain different histones and histone variants. There are entirely different H1 molecules in the MAC and MIC (Allis et al. 1984; Glover et al. 1981; Nabeel-Shah et al. 2020; Qiao et al. 2017). H3 clipping occurs specifically in the MIC (Allis et al. 1979; Wei et al. 2022). H2A.Z and H3.3 are MAC-specific and associated with transcription during cell growth and starvation (Stargell et al. 1993; Wahab et al. 2020). Histones in developing cells exhibit significant acetylation in the MAC (Sharp et al. 2005; Wahab et al. 2020). Although the MIC is transcriptionally silent during the vegetative stage, it is transcribed during the early sexual development stage (Martindale et al. 1985; Mochizuki and Gorovsky 2004; Saettone et al. 2018; Tian et al. 2022).

Histone chaperones are crucial for maintaining chromatin integrity and stability in *T. thermophila*. Disturbance of Nrp1 leads to abnormal mitosis in the MIC and abnormal amitosis in the MAC (Lian et al. 2021). Nrp1 deletion affects the nuclear import of H3 and H3K56ac (Lian et al. 2022). RebL1, a single homolog of human RBBP4/7 proteins, was identified and co-purified with H4 in *T. thermophila* (Nabeel-Shah et al. 2021). However, a comprehensive understanding of RebL1 remains elusive. Here, we found that RebL1 was localized evenly in the MAC, and its subcellular distribution was dynamically changed in the MIC. *REBL1* knockdown inhibited cellular proliferation, leading to MAC swelling and abnormal micronuclear meiosis. RebL1 potentially interacted with a wide range of proteins belonging to multiple chromatin modifying and remodeling complexes. Understanding the function of RebL1 is important for unraveling the molecular mechanisms of the structural integrity of the MAC and MIC during asexual and sexual reproduction in ciliates.

## Materials and methods

### Strain culture and mating

*T. thermophila* B2086 (II), CU428 (VII), and CU427 (VI) were obtained from the National *Tetrahymena* Stock Center (http://tetrahymena.vet.cornell.edu/, Cornell University, Ithaca, NY). Cells were cultured at 30 °C in a super proteose peptone medium (1% proteose peptone, 0.1% yeast extract, 0.2% glucose, and 0.003% EDTA-Fe). For starvation, log-phase cells were washed with 10 mmol/L Tris–HCl (pH 7.4) and resuspended in 10 mmol/L Tris–HCl (pH 7.4) at 30 °C for 16–24 h. The distinct mating type cells were mixed and initiated sexual development.

### Identification of *REBL1*

*REBL1* (TTHERM_00688660) sequences were obtained from the *Tetrahymena* Genome Database (http://www.ciliate.org). DNAMAN was used for aligning amino-acid sequences. Structural and functional domains were identified from the Conserved Domain Database (http://www.ncbi.nlm.nih.gov/Structure/cdd/cddsrv.cgi).

### Protein structure prediction

The RebL1 structure was predicted by the I-TASSER (Iterative Threading ASSEmbly Refinement, https://zhanggroup.org/I-TASSER/) algorithm, which employs a multi-threading method to find structural templates in the Protein Data Bank (PDB). Subsequently, an atomic model was constructed through iterative template-based fragment assembly simulation. The 3D model was then re-threaded by BioLiP to predict the function of the target. The predicted results were visualized using Discovery Studio (http://www.discoverystudio.net/). The results were enhanced using Photoshop 2022.

### Construction of *REBL1*-HA transformants and* REBL1* knockout mutants

The C-terminal sequence (972 bp) and flanking sequence (666 bp) of *REBL1* were amplified by PCR using primers *REBL1*-HA-5F/*REBL1*-HA-5R and *REBL1*-HA-3F/*REBL1*-HA-3R. The amplified fragments were ligated into the pMD-19 T vector, and then the *REBL*1 C-terminal sequence and flanking sequence were digested with *Sac* I/*Not* I and *Xho* I/*Kpn* I, respectively. The digested fragments were ligated with pHA-Neo4. The recombinant fragment was amplified with primer Shoot-*REBL1*-HA-F/Shoot-*REBL1*-HA-R and transferred into *T. thermophila* by biolistic transformation with a biolistic particle delivery system (SCIENTZ, China). Transformants containing the *NEO4* cassette are paromomycin-resistant (Mochizuki 2008; Qiao et al. 2022). The transformants were therefore selected by paromomycin, and the mutants were confirmed by PCR with the primer J-*REBL1*-HA-F/J-*REBL1-*HA*-*R.

The 5ʹ and 3ʹ flanking sequences of *REBL1* were amplified with primers K-*REBL1*-5F/K-*REBL1*-5R and K-*REBL1*-3F/K-*REBL1*-3R, respectively. The fragments were ligated with pMD-19T. The recombinant plasmids were then digested with *Sac* I/*Not* I and *Xho* I/*Kpn* I. The digested fragments were ligated with pNeo4 and digested with the same enzymes. The recombinant plasmid pNeo4-*REBL1* was then digested with *Sac* I/*Kpn* I and transformed into *T. thermophila* by the biolistic particle delivery system. Transformants were selected by paromomycin resistance, and the mutants were confirmed by PCR with the primer J-K-*REBL1*-F/J-K-*REBL1*-R.

### Construction of HA-*REBL1* and HA-truncated *REBL1* mutants

The *REBL1* or truncated *REBL1* were amplified using different primers and then ligated into the pMD-19T vector. After sequencing, the fragments were digested using *Bam*H I/*Sgs* I and subsequently inserted into the pXS75 vector. The constructed plasmid was digested with *Sac* I/*Xho* I, and the resulting fragment was transformed into *T. thermophila* by biolistic transformation using the GJ-1000 (SCIENTZ, Ningbo, China). Subsequently, the mutants were chosen through paromomycin screening.

### Construction of conditionally induced interference mutants

Fragments (500 bp) unique to *REBL1* were amplified using primers RNAi-*REBL1*-1F/RNAi-*REBL1*-1R and RNAi-*REBL1*-2F/RNAi-*REBL1*-2R. The two fragments were digested with *Pst* I/*Sma* I and *Bam*H I/*Pme* I. The phpNeo5 vector digested by the same enzyme was ligated with the digested fragments. The interference plasmid p*REBL1*hpNeo5 was transformed into the MAC of *T. thermophila* by biolistic transformation using the GJ-1000 (SCIENTZ, Ningbo, China). Transformants were chosen by paromomycin resistance. The knockdown efficiency of *REBL1* was confirmed by qRT-PCR with the primer RT-*REBL1*-F/RT-*REBL1*-R.

### RT-PCR and qPCR

RNA was extracted with TRIeasy reagent (Yeasen Biotechnology, Shanghai, China) and converted into cDNA using a Hifair II 1st Strand cDNA Synthesis Kit (Yeasen Biotechnology, Shanghai, China). The cDNA was used for quantitative PCR analysis using a Bio-Rad CFX Connect Real-time System (Bio-Rad), with the threshold series number determined by Bio-Rad CFX Maestro software. The primers employed are listed in Supplementary Table S2. 17S rRNA served as an endogenous control.

### Indirect immunofluorescence staining

The cells (5 mL) were fixed with 20 μL of Schaudinn’s fixative (saturated HgCl_2_: ethanol, 2:1). Then 10 μL of fixed cells were uniformly spread on a poly-L-lysine-coated coverslip. The cells were washed using PBST (0.05% Triton X-100) for 10 min. The cells were blocked using a blocking solution (3% BSA, 10% normal goat serum, and 0.05% Triton X-100 in PBS) for 1 h at room temperature (RT). They were then incubated overnight with HA antibody (1:500 dilution, #3724S, CST, Danvers, MA, USA), γH2AX (1:200, Clone 2F3, BioLegend, USA), and H3K56ac (1:500 dilution; AB_2661786, Active Motif, Carlsbad, CA, USA) at 4 ℃ overnight. The samples were washed three times with PBST (0.05% Triton X-100) and incubated with FITC-conjugated anti-rabbit IgG antibody (1:1000, AQ132F, Millipore, Billerica, MA, USA) or TRITC-conjugated anti-rabbit IgG antibody (dilution ratio of 1:500, AP192R, Millipore, Billerica, MA, USA) for one hour at RT. The samples were stained with 1 μg/mL DAPI for 15 min and observed using a Delta Vision Elite deconvolution microscope system (Applied Precision/GE Healthcare, Boston, Massachusetts, USA).

### Co-immunoprecipitation and mass spectrometry

Cells (1 × 10^7^) were dissolved in 500 μL lysis solution with an inhibitor cocktail (Thermo Fisher Scientific, Waltham, MA, USA) and 50 mmol/L EDTA. After ultrasonic crushing and centrifugation, the supernatant was incubated with 20 μL of packed anti-HA agarose (Thermo Fisher Scientific, Waltham, MA, USA) in a spin column overnight at 4 °C. The sample was centrifuged at 885 *g* for 30 s and washed seven times with TBST (25 mmol/L Tris–HCl, 0.15 mol/L NaCl, pH 7.2, 0.05% Tween-20). The HA-tagged protein was eluted with 25 μL non-reducing sample buffer and boiled for 5 min. Then, 5.5, 6.5, and 12 μL of the samples were used for Western blot, silver staining, and mass spectrometry, respectively.

Trypsin was added to the sample (mass ratio of 1:50) after reduction and alkylation, and the samples were incubated at 37 °C for 20 h. The sample was desalted, lyophilized, and then redissolved in 0.1% FA solution before being kept at −20 °C. Following the calibration of the column using 95% liquid A (0.1% formic acid aqueous solution), an automated sampler was used for inserting the sample into the trap column. Following each complete scan, twenty fragments were collected (MS2 Scan). Proteome Discoverer1.4 software was used to search the corresponding database for the original file of the mass spectrometry test. The fold change of peptide counts for each individual interaction was computed as the peptide counts in the bait divided by the peptide counts of the same prey in the control purifications (zero counts were replaced by 0.1). Mass spectrometry data obtained after immunoprecipitation of wild-type (WT) cells without HA-tag was used as a control. The proteins with a RebL1-HA/WT ratio of more than 40 (vegetative) or 20 (8 h of conjugation) were defined as proteins that have a specific interaction with RebL1.

## Results

### Characterization of histone chaperone RebL1

**Fig. 1:**
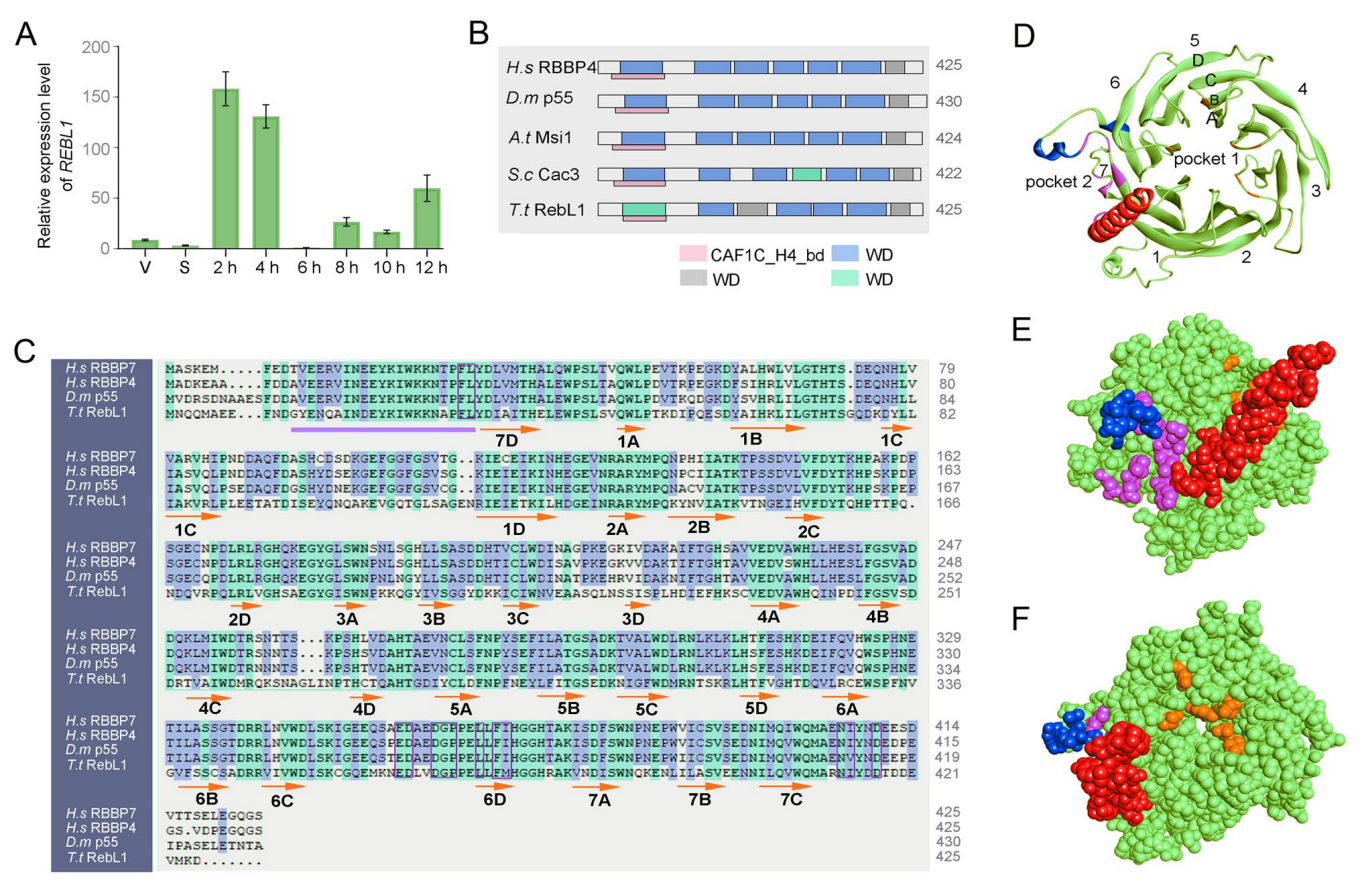
Bioinformatics analysis of *REBL1* from *T. thermophila*. **A** Expression profile of *REBL1*. The samples were collected during vegetative growth (V), starvation (S), and the sexual development stage (2, 4, 6, 8, 10, and 12 h). **B** The conserved domain of RebL1. RebL1 contains the CAF1C_H4_bd domain (pink rectangle) and the WD domain, which consists of seven WD40 repeats. The blue rectangle indicates the fully conserved WD40 domain ending with WD, FD, and YD. The gray rectangle indicates WD ending with conserved tryptophan plus any amino acid (WX). Green indicates WD-like domains that do not end in WD, FD, YD, or WX. *H.s* RBBP4 (*Homo sapiens* RBBP4, Q09028); *D.m* p55 (*Drosophila melanogaster* p55, Q24572); *A.t* Msi1 (*Arabidopsis thaliana* Msi1, O22467); *S.c* Cac3 (*Saccharomyces cerevisiae* Cac3, P13712); *T.t* RebL1 (*Tetrahymena thermophila* RebL1, I7MMT8).** C** Alignment of *T. thermophila* RebL1 with *H.s* RBBP7, *H.s* RBBP4, and *D.m* p55. The secondary structure is illustrated with orange arrows for β-sheet and purple lines for α-helix. The number and letter on the arrow indicate the position of the folded sheet. The binding sites with histone 4 are denoted by the purple boxes. *H.s* RBBP7 (*Homo sapiens* RBBP7, Q16576). **D** Cartoon diagram of RebL1 tertiary structure. Blue indicates the PP loop, red represents the N-terminal alpha helix, pink indicates the binding site to H4, and orange indicates the binding site to H3. Pocket 2 is the binding pocket to histone 4, located between the N-terminal alpha helix and the PP loop. Pocket 1 is the binding pocket to histone 3. **E** The tertiary structure RebL1 in CPK presentation style. The content indicated by different colors is consistent with diagram D. **F** The tertiary structure of RebL1 is presented in the form of CPK with a different orientation from the E diagram. The content indicated by different colors is consistent with diagram D

*REBL1* (TTHERM_00688660) had low expression in the vegetative growth and starvation stage and high expression during the sexual reproduction stage, reaching the highest expression 2 h after mixing (Fig. 1A). The expression profile of *REBL1* resembled that in microarray expression data (http://tfgd.ihb.ac.cn). RebL1 is an evolutionally conserved WD40-repeat family protein (Nabeel-Shah et al. 2021). It possesses seven WD40 repeats and forms a β-propeller conformation, which contributes to protein–protein interactions (Fig. 1B, C). The first WD40 repeats of RebL1 were conserved with terminal dipeptide (WD, FD, and YD), the second WD40 repeats contained WX dipeptide (Trp or random amino acid), and the last WD40 repeats ended without WD, FD, YD, or WX (Fig. 1B). Each WD40 repeat contained four β folds (Fig. 1D). In accordance with the structure of RBBP4/RBBP7, the structure of RebL1 was predicted to form a stabilized circular structure. The binding sites of RebL1 with H3 and H4 were identified: pocket 1 binds H3, and pocket 2 binds H4 (Fig. 1D–F).

### Dynamic localization of RebL1

**Fig. 2:**
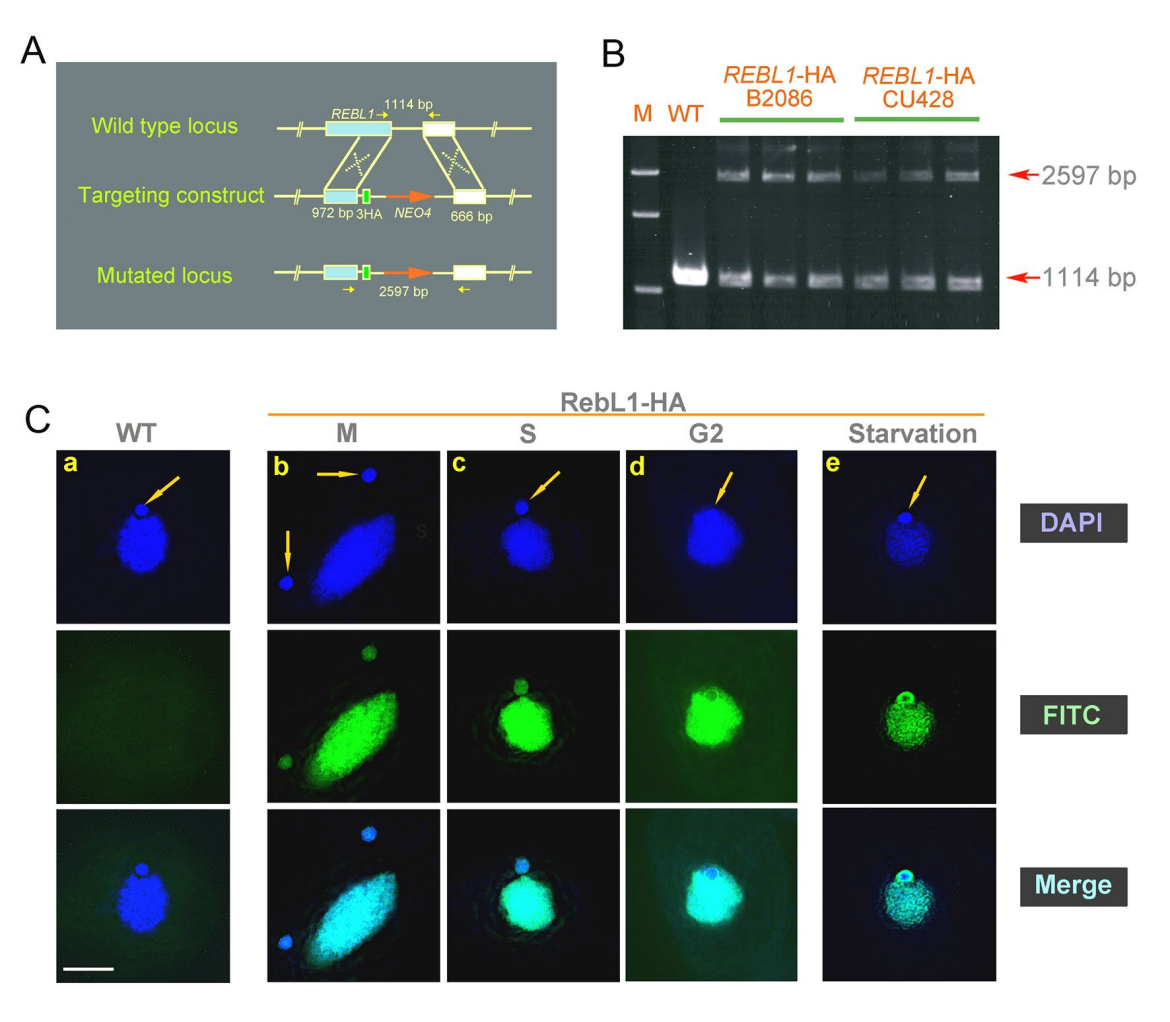
Dynamic localization of RebL1 during vegetative growth and starvation stage. **A** Schematic of homologous recombination of p*REBL1*-HA. The cyan rectangle represents *REBL1* and 5' homologous arm. The green rectangle signifies HA-Tags. The orange arrow indicates the *NEO4* cassette. The white rectangle denotes the 3' homologous arm. The yellow arrows indicate the position of the primer used to identify the homologous arm. **B** Homologous recombination substitution in RebL1-HA mutant strain. M denotes the marker. Arrows indicate mutant loci (2597 bp) and WT loci (1114 bp). **C** Localization of RebL1-HA during vegetative growth and starvation. Arrows indicate MIC. a, WT; b, MAC amitosis; c, S phase of MIC; d, G2 phase of MIC; e, starvation for 24 h (*n* = 20). Scale bar, 10 µm

The Cac3/RBBP4 localizes in both the cytoplasm and nucleus in *S. cerevisiae* (Johnston et al. 2001). To investigate the dynamic localization of RebL1, the recombinant plasmid p*REBL1*-HA was constructed and transformed into *T. thermophila* (Fig. 2A). The transformants were identified by PCR (Fig. 2B). RebL1-HA localized in the MAC and MIC during the vegetative growth and starvation stages (Fig. 2Ca–e) and formed a ring structure around the MIC during the micronuclear G2 phase and starvation stage (Fig. 2Cd–e).

**Fig. 3:**
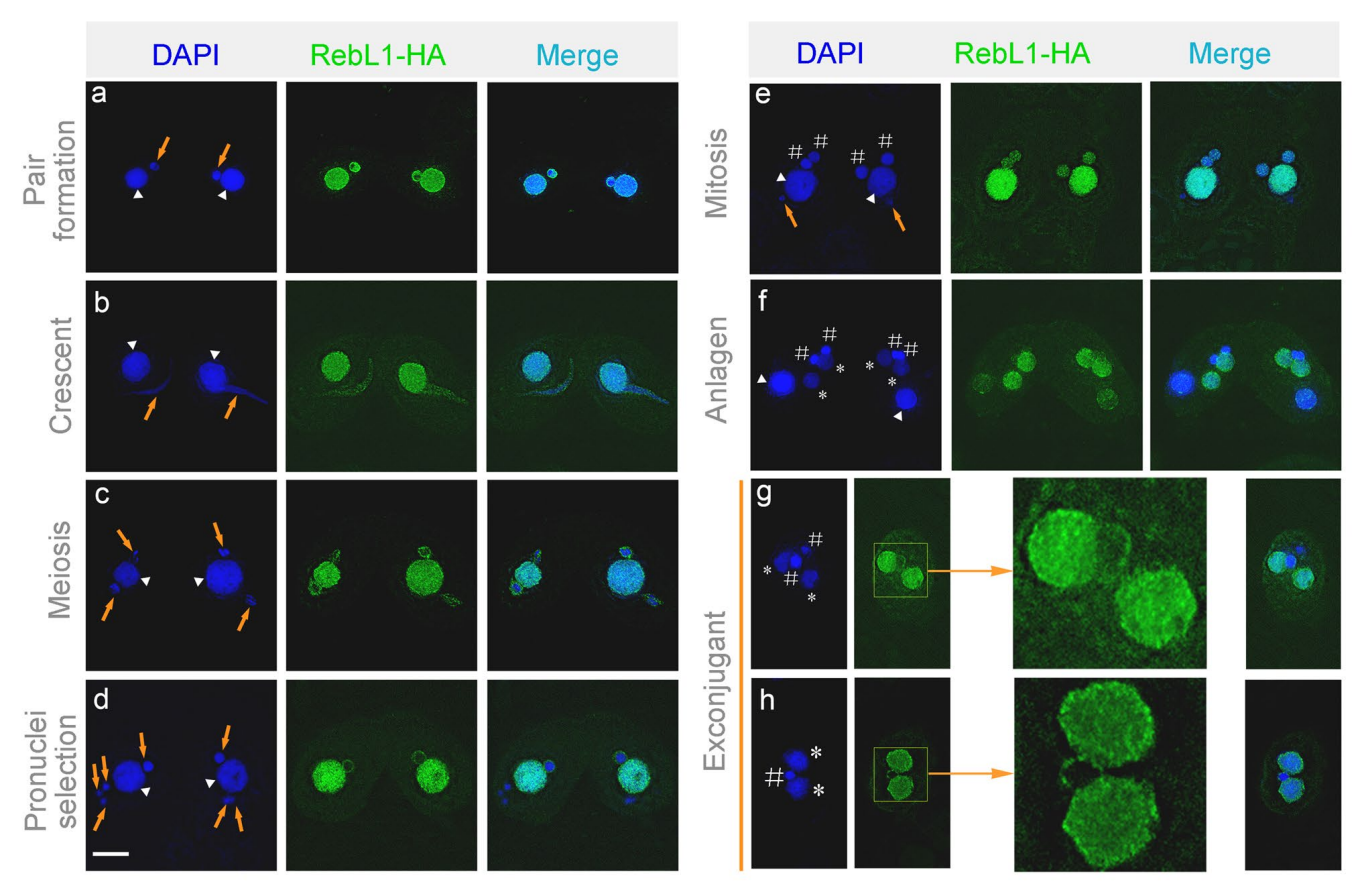
Localization of RebL1-HA during sexual reproduction. a, pair formation; b, crescent; c, meiosis; d, pronuclei selection; e, mitosis; f, anlagen; g, exconjugant with two MACs and two MICs; h, exconjugant with two MACs and one MIC. Triangles indicate parental MACs, arrows indicate MICs, # indicates the zygotic nucleus, and * indicates new MACs (*n* = 20 per period). The area inside the box is magnified 3.6 times in the right margin. Scale bar, 10 µm

Although the transcription level of *REBL1* varied during the sexual development stage (Fig. 1A), Western blotting analysis showed that RebL1 maintained a stable expression level (Supplementary Fig. S1). The protein expression profile indicated that RebL1 is stable and could function as a scaffold protein during the conjugation stage. RebL1-HA localized in parental MACs at the early sexual development stage and transferred into new MACs at the early anlagen stage (Fig. 3a–h). However, the signal in parental MACs weakened at the late anlagen stage (Fig. 3f). RebL1-HA also localized in meiotic and mitotic MICs and disappeared in degraded meiotic products (Fig. 3d). Furthermore, it formed a ring structure around early meiotic MICs (Fig. 3a) and functional gametic nuclei (Fig. 3d).

Previous studies have reported that RBBP7 binds directly to H4 (Murzina et al. 2008) and a potential interaction between RebL1 and H4 has been identified in *Tetrahymena* (Nabeel-Shah et al. 2021). To further investigate the relationship between RebL1 and H4, four recombinant plasmids harboring an N-terminal HA-tag, i.e., pOE-*REBL1*, pOE-*REBL1*^*TrN89*^ (truncated CAF1C_H4_bd domains), pOE-*REBL1*^*TrN35*^ (truncated N-terminal histone binding sites), and pOE-*REBL1*^*TrC*^ (truncated C-terminal histone binding sites), were created (Supplementary Fig. S2A, B). The target genes were regulated by *MTT1* promoter and induced by Cd^2+^ (Supplementary Fig. S2B). After the plasmids were transformed into *T. thermophila,* the mutant strains of RebL1 were obtained (Supplementary Fig. S2C, D). The HA-RebL1 localized in the periphery of MICs in the G2 phase during growth (Supplementary Fig. S3b). The localization of HA-RebL1 was similar to that of RebL1-HA both during asexual and sexual reproduction (Supplementary Fig. S3b–e). HA-RebL1^TrN89^ localized in the cytoplasm during vegetative growth and conjugation (Supplementary Fig. S3f–i). HA-RebL1^TrN35^ also localized in the cytoplasm (Supplementary Fig. S2j–m). Interestingly, HA-RebL1^TrN35^ transiently imported into the replicating new MACs (Supplementary Fig. S3l). HA-RebL1^TrC^ also localized in the cytoplasm throughout the developmental process (Supplementary Fig. S2n–q). The defect of the H4 binding domains of RebL1 affected its nuclear distribution. Moreover, RebL1 had no predictable classic nuclear localization signal. These findings suggested that the nuclear translocation of RebL1 could be involved in histone H4 binding domains or interaction with H4.

### *REBL1* knockdown affected macronuclear structure and cellular proliferation

**Fig. 4:**
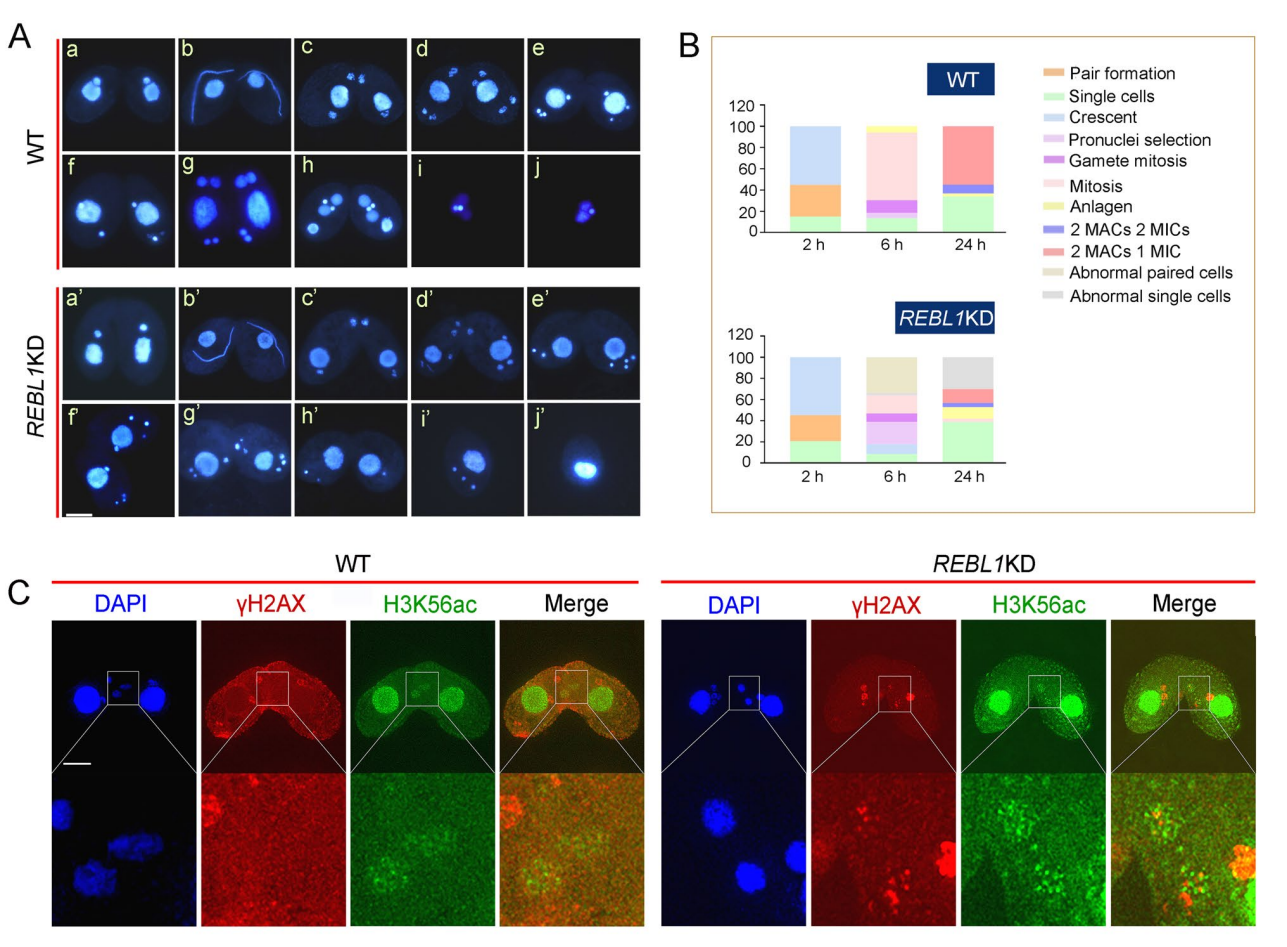
*REBL1* knockdown affected gametic nucleus formation. **A** Nuclear development of WT and *REBL1*KD. a and a’, pair formation; b and b’, crescent; c and c’, meiosis I; d and d’, meiosis II; e and f’, pronuclei selection; f, mitosis I; g, mitosis II; h, anlagen; i, exconjugant with two MACs and two MICs; j, exconjugant with two MACs and one MIC; e’, g’ and h’, abnormal paired cells; i’ and j’, abnormal single cells. Scale bar, 10 µm. **B** Statistics of nuclear development during sexual reproduction in *REBL1*KD (*n* = 300). **C** Co-localization of γH2AX and H3K56ac in WT and mutants. The area inside the box is magnified nine times in the lower margin. Scale bar, 10 µm

RBBP4 and RBBP7 exist together in several transcriptional complexes and play a redundant function during preimplantation development (Xiao et al. 2022). To investigate the function of RebL1, the *REBL1* knockout plasmid was constructed. The *REBL1* knockdown mutants, *REBL1*KDB (mating type II) and *REBL1*KDC (mating type VII) were created (Supplementary Fig. S4A–C). We failed to obtain *REBL1* knockout mutants through phenotype screening under paromomycin selection, which suggested *REBL1* is essential for cell survival. qRT-PCR showed 35.74% and 55.14% reduction of *REBL1* transcripts in the different mutants. The proliferation of *REBL1*KD was similar to that of WT (Supplementary Fig. S4D). During the early conjugation stage, *REBL1* knockdown mutants completed meiosis normally (Fig. 4Aa–d, a’–d’). Among four meiotic products, one is chosen and undergoes mitosis to produce gametic nuclei. At 6 h of conjugation, 63.62% of the WT cells were undergoing micronuclear mitosis (Fig. 4Af, g, B), however, only 17.16% of the mutant cells performed micronuclear mitosis, and 33.66% of mutant cells were aberrant at this stage (Fig. 4Ae’, g’, B). Finally, 55.07% of the WT cells developed into exconjugants with two MACs and one MIC. In contrast, 13.09% of mutants developed into exconjugants with two MACs and one MIC at 24 h of mixing (Fig. 4A, B). Furthermore, γH2AX and H3K56ac were investigated to determine whether DSBs were repaired after meiosis. In the WT, H3K56 of the selected pronucleus was acetylated and the signal of γH2AX disappeared with the repair of the DNA damage. However, the γH2AX signal and H3K56ac modification were maintained in the *REBL1*KD strain, which indicated defective DNA repair in the mutants (Fig. 4C).

**Fig. 5:**
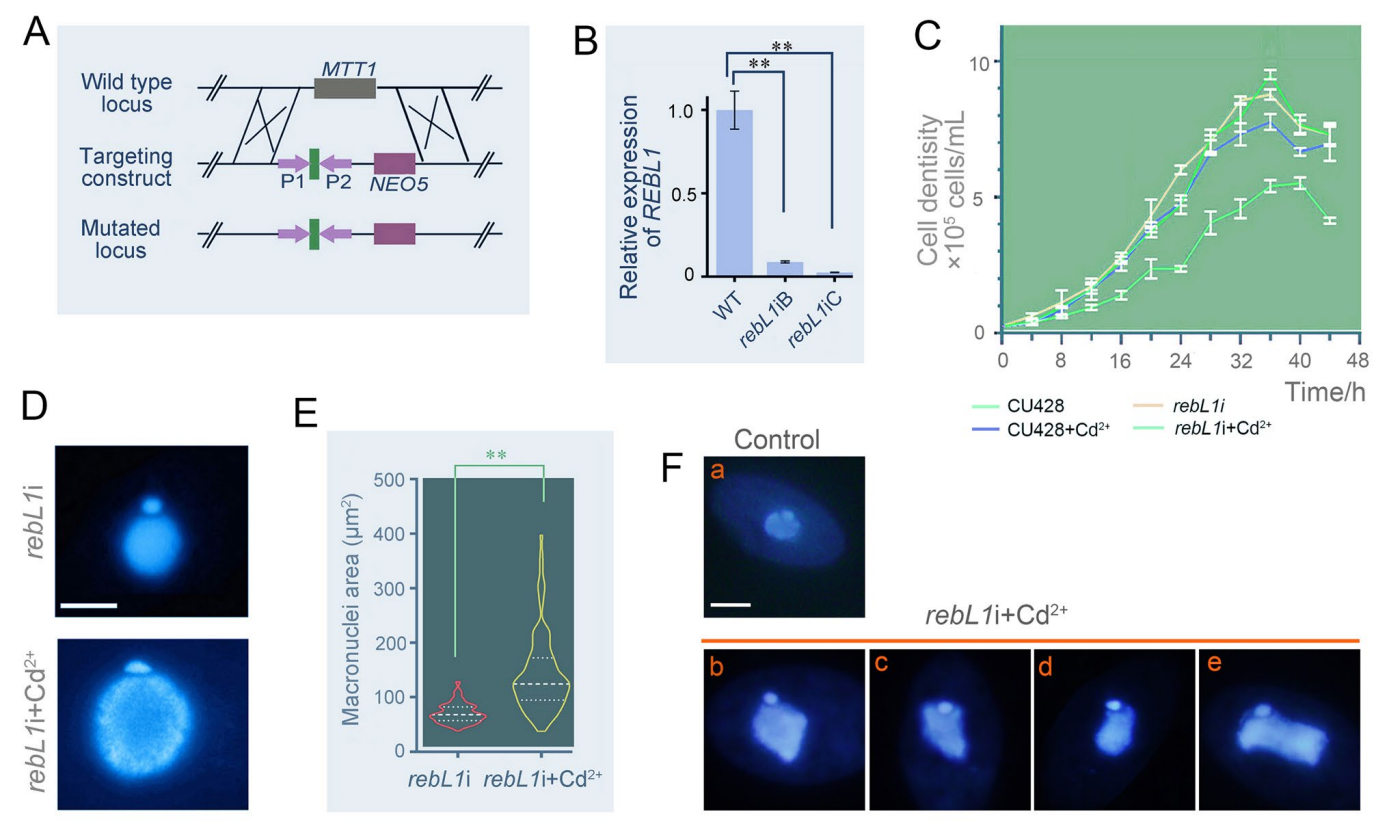
*REBL1* knockdown by RNAi affected the proliferation of *T. thermophila.* **A** Schematic of *rebL1*i homologous recombination. The *MTT1* is represented by the gray rectangle, and the two segments P1 and P2 in *REBL1* are indicated by the purple arrows. The short linker is depicted by the green rectangle, and the *NEO5* cassette is denoted by the purple rectangle. **B** Relative expression of *REBL1* in *rebL1*i mutants and WT. Cells were induced under 0.5 μg/mL Cd^2+^ for 96 h. T-test was applied for significance analysis (** *P* < 0.01). **C** Proliferation of *rebL1*i mutants and WT. CU428, *rebL1*iC, and CU428 + Cd^2+^ were used as controls. CU428 + Cd^2+^ and *rebL1*iC + Cd^2+^ were induced for 96 h at 0.5 μg/mL Cd^2+^. **D** Nuclear morphology of *rebL1*i mutants. Cells were fixed with formaldehyde and stained with DAPI. Scale bar, 10 µm. **E** Macronuclear area in *rebL1*i. *rebL1*i mutants were cultured with and without Cd^2+^ for 96 h and then fixed with formaldehyde. The area of large nuclei was measured using ImageJ (*n* = 100). *T* test was applied for significance analysis (*P < 0.05; **P < 0.01). **F** Morphology of MAC in *rebL1*i mutants during starvation. Cells were cultured in SPP medium containing 0.5 μg /mL Cd^2+^ for 96 h and then starved in Tris–HCl with 0.25 μg/mL Cd^2+^ for 24 h. Cells were then fixed with formaldehyde and DAPI staining was performed (*n* = 100). Scale bar, 10 µm

To further investigate the stage-specific function of RebL1, conditional knockdown *rebL1*i was created (Fig. 5A). RNAi was induced by adding Cd^2+^ to the mutant (Howard-Till et al. 2013). The expression levels of the *rebL1*iB (mating type II) and *rebL1*iC (mating type VII) decreased by 91.2% and 97.44% when exposed to 0.5 μg/mL Cd^2+^ for 96 h, respectively (Fig. 5B). Proliferation of *rebL1*i mutants decreased compared to that of WT (Fig. 5C). Furthermore, mutants had larger MACs (Fig. 5D, E). After being starved for 24 h, the MACs of 41.75% mutants and 4% WT became irregular and abnormal (Fig. 5Fb–e). These findings suggested that *REBL1* knockdown affects macronuclear structure and cellular proliferation.

### *REBL1* knockdown abolished sexual development

**Fig. 6:**
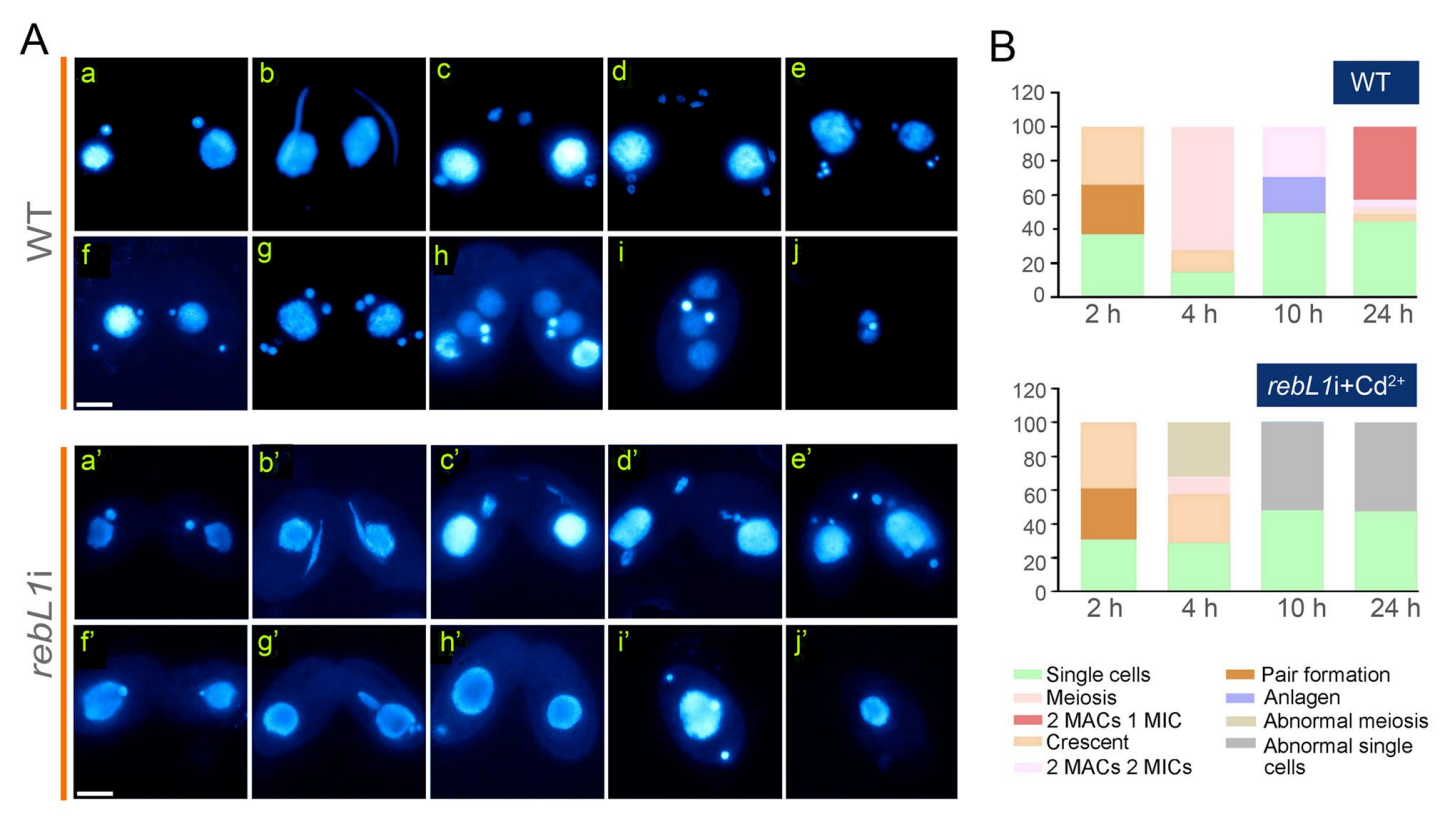
*REBL1* knockdown affected sexual reproduction of *Tetrahymena*. **A** Sexual reproductive development of *rebL1*i and WT induced with Cd^2+^. 0.1 μg/mL Cd^2+^ was added during starvation and subsequently increased to 0.25 μg/mL after 2 h of pairing. a and a’, pair formation; b and b’, crescent; c, meiosis I; d, meiosis II; e, pronuclei selection; f, mitosis I; g, mitosis II; h, anlagen; i, exconjugant with two MACs and two MICs; j, exconjugant with two MACs and one MIC; c’–e’, abnormal meiosis; f’–h’, abnormal paired cells; i’ and j’, abnormal single cells. Scale bar, 10 µm. **B** Statistics of nuclear development during sexual reproduction in *rebL1*i strains and WT (*n* = 300 per period)

The knockdown of *REBL1* inhibited cellular proliferation during vegetative growth. To investigate the role of RebL1 during conjugation, the mutants were induced with 0.1 μg/mL Cd^2+^ for 24 h during starvation. Then, the different mating type cells were mixed. The MICs began meiosis and stretched normally (Fig. 6Aa’, b’), and in *rebL1*i, meiosis was abnormal in 31.97% of conjugants at 4 h, i.e., the micronuclear chromosome was lost (Fig. 6Ac’–e’, B) and gametic nuclei failed to form (Fig. 6Af’–h’). Of the mating *rebL1*i mutants, 52.35% separated abnormally and formed abnormal single cells (Fig. 6Ai’–j’, B)*.* At 24 h after mixing, 42.75% of cells in the WT completed sexual reproduction, however, the mating mutants failed to develop into exconjugants with two MACs and one MIC (Fig. 6B).

### Overexpression of *REBL1* affected cellular proliferation and sexual development

Overexpression of RBBP4 is found in several cancer types such as thyroid carcinomas (Pacifico et al. 2007). The patients who expressed high RBBP4 have shorter overall survival times (Hart et al. 2021; Li et al. 2019; Zheng et al. 2013). In the present study, *REBL1* was overexpressed under 0.5 μg/mL Cd^2+^ induction to further investigate the function *REBL1* played. *REBL1* was upregulated by 61 and 284-fold in OE-*REBL1*B and OE-*REBL1*C mutants, respectively (Supplementary Fig. S5A). The overexpression of *REBL1* inhibited the proliferation of mutant cells (Supplementary Fig. S5B). During sexual reproduction, only 7.12% of cells developed into exconjugants with two MACs and one MIC, and 47.99% of cells were abnormal (Supplementary Fig. S5C, D). The overexpression of *REBL1* not only inhibited cellular proliferation but also affected sexual development in *T. thermophila*.

### RebL1 interacted with different chromatin-associated proteins

**Fig. 7:**
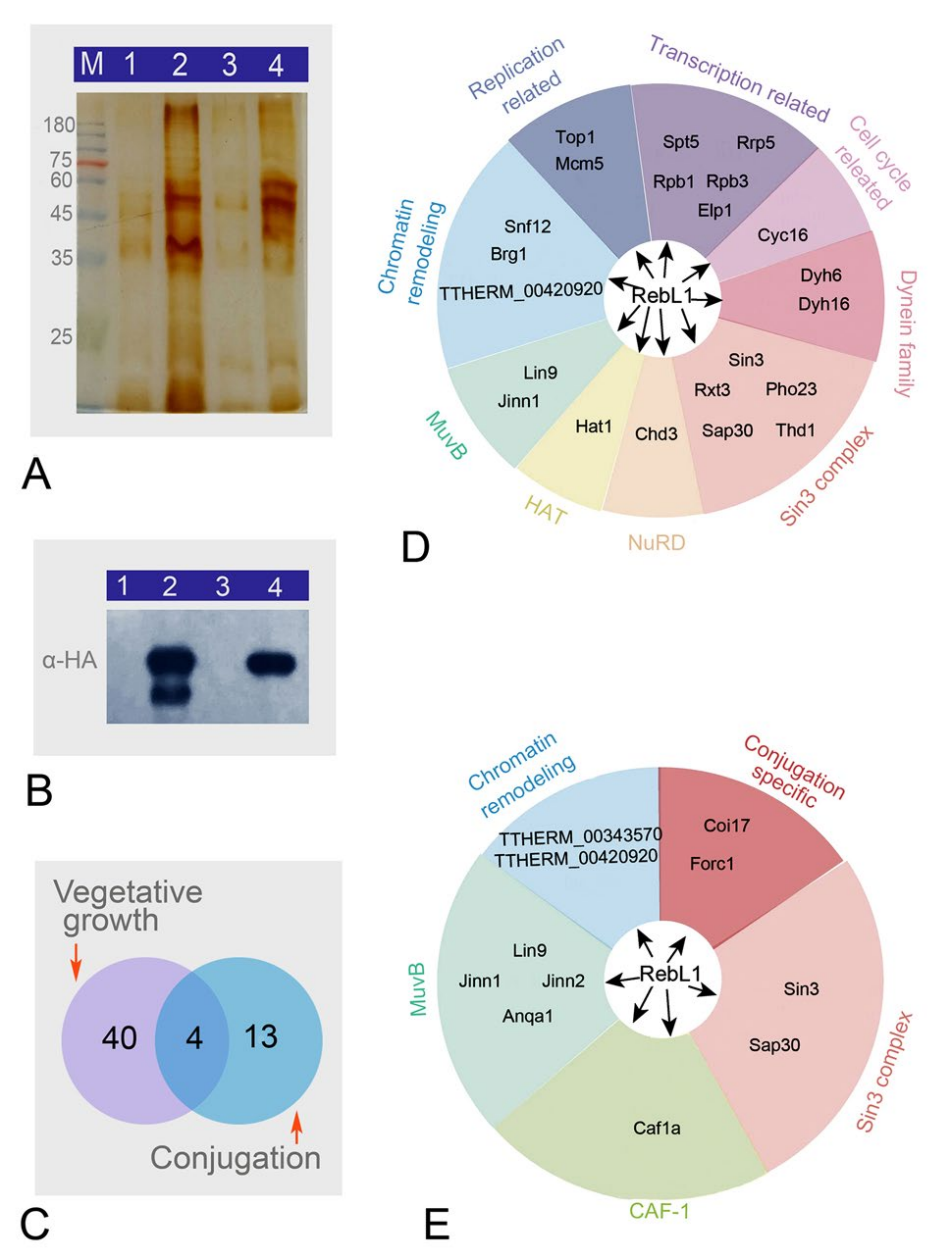
Identification of proteins interacting with RebL1. **A** Silver-stained band of cell lysate after passing through HA-tag gel column. M, protein pre-stained marker; 1, WT sample during vegetative growth; 2, RebL1-HA sample during vegetative growth; 3, WT sample during conjugation 8 h; 4, RebL1-HA sample during conjugation 8 h. **B** Western blot of cell lysate after passing through HA-tag gel column. **C** Venn diagrams illustrate unique and shared proteins interacting with RebL1 during vegetative growth (purple) and conjugation (blue). **D** RebL1-HA interaction network during vegetative growth. **E** RebL1-HA interaction network during conjugation

RBBP4/7 have been shown to be components of the CAF-1, HAT1, NURF, and NuRD complex. Nabeel-Shah et al. (2021) showed that RebL1 interacts with diverse chromatin-associated proteins during the vegetative growth and sexual developmental stages by expressing RebL1 with a C-terminal FZZ epitope tag. All the RebL1 interaction partners identified during vegetative growth are also detected in the conjugating 5 h post-mixing *Tetrahymena*. At conjugation 8 h, degradation of the parental MAC is initiated, the new MAC begins to form, and the genome of the new MAC undergoes replication and rearrangement (Austerberry et al. 1984; Xu et al. 2021). RebL1 showed strong localization signals in the newly developing MAC (Fig. 3f). To study the function and the potentially interacting partners of RebL1 after 8 h of conjugation, co-immunoprecipitation and affinity purification-mass spectrometry (AP-MS) analysis was performed during the vegetative phase and 8 h post-mixing (Fig. 7A, B; Supplementary Tables S3, S4). During the vegetative growth stage, 44 proteins that potentially interacted with RebL1 were identified, including different chromatin-associated components. I: type B histone acetyltransferase Hat1; II: histone deacetylase Thd1/Rpd3, TTHERM_00450950/Sin3, TTHERM_00476650/Pho23, Sap30, TTHERM_00992830/Rxt3 (components of Sin3/HDAC histone deacetylation complex); III: Chd3, a component of the nucleosome remodeling and histone de-acetylation NuRD; IV: Lin9 and Jinn1, components of the MuvB transcriptional regulatory complex; V: Dyh6 and Dyh16, components of the dynein family; VI: proteins related to DNA replication and transcription, chromatin remodeling, and nuclear import (Fig. 7C, D).

Seventeen potential interaction partners were obtained at conjugation 8 h, which included components of Sin3, CAF-1, MuvB complexes, proteins associated with chromatin remodeling, and proteins specific to conjugation (Fig. 7C, E). The MuvB complex contained Anqa1, Lin9, and Jinn1. The CAF-1 complex included the large subunit Caf1a. The chromatin remodeling-related proteins included TTHERM_00343570 (homologous to INO80 ATPase in yeast). The conjugation stage-specific proteins that potentially interacted with RebL1 included Coi17 (conjugation-induced 17), and Forc1 (friend of RebL1 in conjugation). The proteins potentially interacting with RebL1 that were common to the vegetative growth and sexual reproduction stages were Sin3, Sap30, Jinn1, and Lin9. However, components of PRC2 and NURF complexes were absent in the RebL1 interaction partners during the growth and conjugation stages.

### Downregulated expression of genes involved in chromatin organization and transcription

**Fig. 8:**
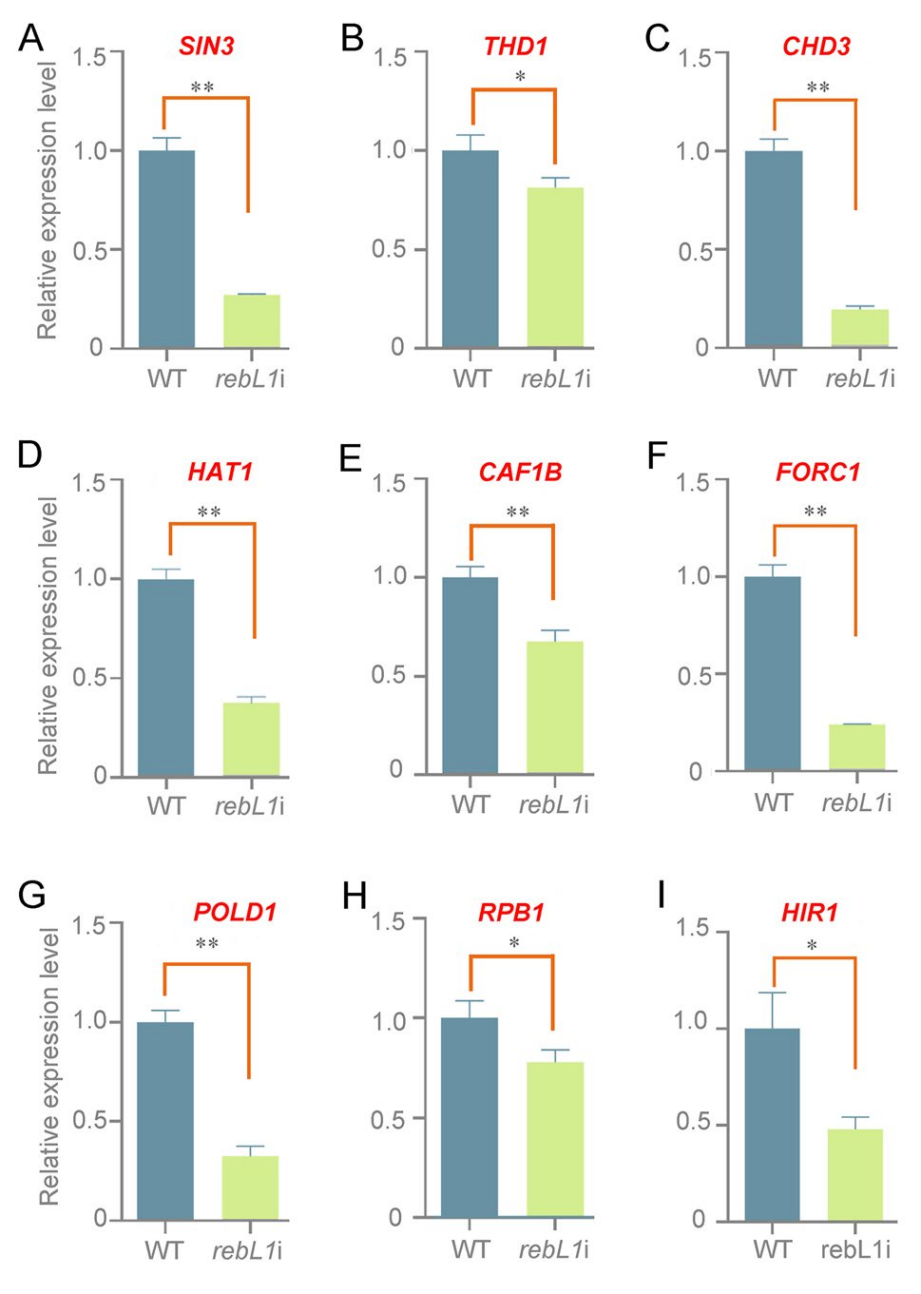
Relative expression levels of genes involved in *REBL1* in *rebL1*i mutants. **A**–**I** Relative expression levels of *SIN3*, *THD1*, *CHD3*, *HAT1*, *CAF1B*, *FORC1*, *POLD1*, *RPB1*, and *HIR1*. *T* test was applied for significance analysis (*P < 0.05; **P < 0.01)

Previous studies have shown that the expression of *RAD51*, *ANQA1*, and *LIN9* decreased with *REBL1* reduction (Nabeel-Shah et al. 2021). RebL1 is associated with various complexes that participate in chromatin assembly, modification, and remodeling (Nabeel-Shah et al. 2021). To further investigate the expression regulation of specific genes, expression levels of the genes involved in chromatin organization and transcription were examined in *REBL1* knockdown mutants. The expression levels of *SIN3*, *THD1*, *CHD3*, *HAT1*, *CAF1B*, *FORC1*, *POLD1*, and *RPB1* were downregulated in the *rebL1*i mutants (Fig. 8A–H). The expression of *HIR1*, a key factor for non-replication-coupled nucleosome assembly, was also down-regulated in *rebL1*i (Fig. 8I), suggesting that the downregulation of *REBL1* could affect non-replication-coupled nucleosome assembly and replication-coupled nucleosome assembly.

## Discussion

In eukaryotes, the CAF-1 complex is a highly conserved histone chaperone that functions in nucleosome assembly during DNA replication (Ransom et al. 2010; Winkler et al. 2017). Here, we showed that RebL1 dramatically localizes in the functional MAC and MIC and is required for cellular vegetative growth and sexual reproductive development in *Tetrahymena*.

In *Plasmodium falciparum*, PfRbAp46/48 (RBBP7/4 homologous) localizes at the nuclear periphery during the ring stage and overlaps with chromatin during the trophozoite and schizont stages (Kaushik et al. 2020). In *C. elegans*, RbAp46^LIN-53^ (RBBP7 homologous) localizes to the nucleus during interphase and is present at the centromere during metaphase, but is absent during anaphase and telophase (Lee et al. 2016). We found that RebL1 localized in the actively transcriptional MAC during vegetative growth and the sexual developmental stage in *T. thermophila* (Figs. 2C, 3). It also localized in the replicating MIC during the S phase and transferred to the periphery of the MIC during the G2 phase (Fig. 2Cc, d). RebL1 had a weak signal in crescent MICs (Fig. 3b), which perform DNA double-strand breaks and repair. Following the selection of meiotic products, the selected product goes through post-meiotic mitosis, and the remaining three nuclei degenerate (Loidl 2021). The signal of RebL1 was present in the selected pronuclei, which implied that it might participate in gametic DNA replication and chromatin remodeling (Fig. 3d). The gametic nuclei fuse to form a zygotic nucleus which divides twice to produce four nuclei, two of which develop into new MACs (Cole and Sugai 2012; Slade et al. 2011). The new MACs undergo DNA replication and chromatin remodeling (Cole and Sugai 2012; Doerder and Debault 1975). A strong signal of RebL1 occurred in the new MACs, which indicated that RebL1 might be related to DNA replication or genome rearrangement (Fig. 3f). The nuclear periphery is a repressive compartment of nucleus clustering inactive genes. RebL1 in this region might be involved in transcription repression or chromatin remodeling. RebL1-FZZ localizes in the MAC and micronuclear localization disappears with changes in the cell cycle (Nabeel-Shah et al. 2023). However, we found that the signal of RebL1-HA did not disappear in MIC, rather it performed positional shifts during the cell cycle.

RBBP4 is essential for preserving the identity of mouse embryonic stem cells (mESCs) and its loss enhances the transition from mESCs to trophoblast cells (Ping et al. 2023). Furthermore, RBBP4 acts as an essential barrier to prevent the induction of the pluripotent-to-totipotent cell fate transition and plays a significant role in heterochromatin assembly. The depletion of RBBP4 leads to the activation of a cluster of transposable elements (Ping et al. 2023). The loss of RBBP4 results in delayed S-phase development and slower DNA synthesis in chicken DT40 B cells (Satrimafitrah et al. 2016). Deletion of RBBP7 in mouse ovaries inhibits meiotic chromosome deacetylation and leads to chromosome misalignment and spindle abnormalities during meiosis (Balboula et al. 2014). In *C. elegans*, Lin53 (RBBP4 homologous) depletion affects the lifespan of the organism, leading to premature death (Müthel et al. 2019). *REBL1* knockdown affected cellular proliferation, macronuclear structure, and gamete nucleus formation in *Tetrahymena*. We hypothesize that *REBL1* knockdown resulted in aberrant deacetylation of histones in the MAC. At the same time, the knockdown of *REBL1* may disrupt the stability of the CAF-1 complex, leading to aberrant chromatin assembly, loose chromatin structure, and abnormal meiosis or mitosis of MICs. Deletion of RBBP4, p150, and p60 in vertebrates all drive mitotic abnormalities (Satrimafitrah et al. 2016; Takami et al. 2007). Temozolomide-induced γH2AX foci are higher in RBBP4 mutant cells (Kitange et al. 2016). Simultaneous silencing of RBBP4 and RBBP7 increases in H2AX focus-containing primary human fetal fibroblast cells (Pegoraro et al. 2009). In selected pronuclei, the γH2AX signal was abnormally maintained in the *REBL1* knockdown mutants (Fig. 4C). The gametic nuclei failed to form and sexual development was abolished in *Tetrahymena*.

The deacetylation and acetylation of histones determine the acetylation state of H3 and H4. Deacetylation of histones by the histone deacetylase complex Sin3 is one of the primary mechanisms involved in transcriptional repression in eukaryotes. We found that RebL1-HA interacted with the Sin3 complex during vegetative growing stages (Fig. 7D). In eukaryotes, most of the repression activity of the Sin3 complex is attributed to the histone deacetylase activity of Rpd3. The Sin3 complex is thought to mediate transcriptional repression through the gene-specific deacetylation of histones (Bernstein et al. 2000; Silverstein and Ekwall 2005). In budding yeast cells, Sin3 forms large and small complexes with Rpd3. Histones at promoter regions are deacetylated by the Rpd3L complex. On the other hand, the Rpd3S complex suppresses intragenic transcription start by targeting transcribed areas. (Sardiu et al. 2009). Therefore, RebL1 could dynamically regulate the acetylation of histones by different complexes in the MAC. Polycomb repressive complex 2 (PRC2) is the major methyltransferase for H3K27 methylation. In mammals, the PRC2 complex is made up of EED (extrasex combs [ESC, EED]), EZH2 (enhancer of zeste [E(z), EZH2]), SUZ12 (suppressor of zeste 12 [Su(z)12]), and RBBP4 (Deevy and Bracken 2019). In *Drosophila*, p55 is also present in the PRC2 complex. In *Paramecium tetraurelia*, PtCAF1 (RBBP4 homologous) is present in the PRC2 complex which functions in the deletion of internally eliminated sequences (Ignarski et al. 2014). However, RebL1 is absent in the PRC2 complex in *Tetrahymena* (Supplementary Table S1). These findings indicated that RebL1 and the PRC2 complex separated during the early evolution of *Tetrahymena*. RebL1 interacted with Caf1a during the sexual development stage. In *Schizosaccharomyces pombe* and *S. cerevisiae*, histone H3 deposits on the DNA that is being replicated or repaired by CAF-1 and HIR1 (Choi et al. 2005; Li et al. 2012; Pile et al. 2002; Sharp et al. 2005; Winkler et al. 2017; Yadav et al. 2017). CAF-1 depletion in Epstein-Barr virus-positive host cells causes loss of both H3.1 and H3.3 (Siddaway et al. 2022; Zhang et al. 2020). Reduction of *REBL1* led to the downregulation of *HIR1* expression, which might indirectly affect non-replication-dependent nucleosome assembly.

Histone acetylation influences gene expression and chromatin state. In yeast, Hat1 catalyzes the acetylation of newly synthesized histones. Furthermore, Hif1 binds to acetylated histone H4 in a Hat1/Hat2-dependent manner (Ai and Parthun 2004). Hat2/RBBP4 stimulates Hat1 catalytic activity and increases the specificity toward H4K12 (Ai and Parthun 2004; Poveda et al. 2004; Yue et al. 2022). Hat2/RBBP4 functions as a link connecting Hif1 with Hat1, and Hif1 with H4. Acetylation of histone H4 is maintained by the RBBP7-Hat1 complex, which is necessary for the deposition of the histone H3 variant, CENP-A, on centromeres (Kaushik et al. 2020). Previously, we have found that Hif1/Nrp1 disruption leads to abnormal mitosis and amitosis and affects the nuclear import of H3 and H3K56ac (Lian et al. 2021, 2022). *REBL1* knockdown affected expression levels of the genes involved in chromatin organization and transcription (Fig. 8). We propose that *REBL1* knockdown affects histone acetyltransferase Hat1 expression and activity in the cytoplasm and disrupts the histone deacetylase Rpd3 complex. Furthermore, the overexpression of *REBL1* also affected cellular proliferation and sexual reproduction in *Tetrahymena*. These findings underscore the essential role of normal *REBL1* expression during asexual and sexual reproduction. Taken together, these findings suggest that RebL1 is required for macronuclear structure stability and gametogenesis in *T. thermophila.* The present study provides important insights into the functional significance of RebL1 and adds to our understanding of transcriptional regulation and chromatin remodeling processes in ciliates. This knowledge may also have broader implications for our understanding of chromatin dynamics and nuclear organization in other eukaryotic organisms.

### Supplementary Information

Below is the link to the electronic supplementary material.

Supplementary file1 (ZIP 8227 KB)

Supplementary file2 (XLSX 45 KB)

Supplementary file3 (XLSX 17 KB)

Supplementary file4 (DOCX 1255 KB)

## Data Availability

All relevant data are within the paper and its additional files. The data used to support the findings of this study are available upon reasonable request.
