# Supplementary material for: RebL1 is required for macronuclear structure stability and gametogenesis in Tetrahymena thermophila
Source: Mar Life Sci Technol. 2024 Mar 26;6(2):183–97. doi: 10.1007/s42995-024-00219-z (PMC11136921; doi:10.1007/s42995-024-00219-z)
Supplement: Supplementary file 1 — Supplementary file1 (ZIP 8227 KB) [file 42995_2024_219_MOESM1_ESM.zip › 42995_2024_219_MOESM1_ESM/Supplementary materials.docx]

**RebL1 is required for macronuclear structure stability and gametogenesis in *Tetrahymena thermophila***

Huijuan Hao^1^ · Yinjie Lian^1^ · Chenhui Ren^1^ · Sitong Yang^1^ · Min Zhao^1^ · Tao Bo^1^ · Jing Xu^1,2,*^ · Wei Wang^1,3,*^

1 Key Laboratory of Chemical Biology and Molecular Engineering of Ministry of Education, Institute of Biotechnology, Shanxi University, Taiyuan 030006, China.

2 School of Life Science, Shanxi University, Taiyuan 030006, China.

3 Shanxi Key Laboratory of Biotechnology, Taiyuan 030006, China.

*Corresponding author:

Jing Xu: [xujing@sxu.edu.cn](mailto:xujing@sxu.edu.cn,); Wei Wang: gene@sxu.edu.cn, ORCID: 0000-0002-1595-9797

Supplemental Information

Supplemental Figs. S1-S5

Supplemental Tables S1-S2


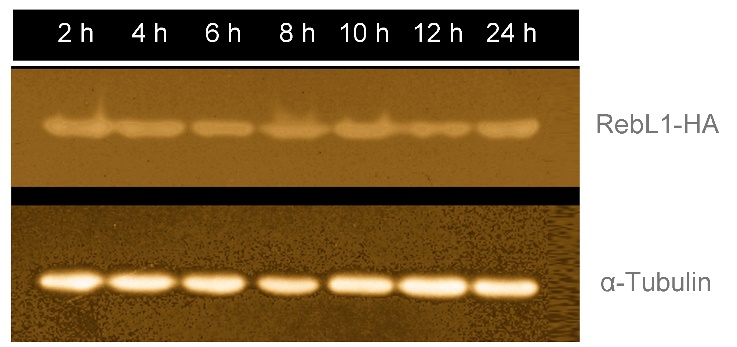


**Fig. S1** Expression analysis of RebL1-HA by Western blotting. The samples were collected at 2 h, 4 h, 6 h, 8 h, 10 h, 12 h, and 24 h during conjugation. The α-tubulin was used as a loading control.


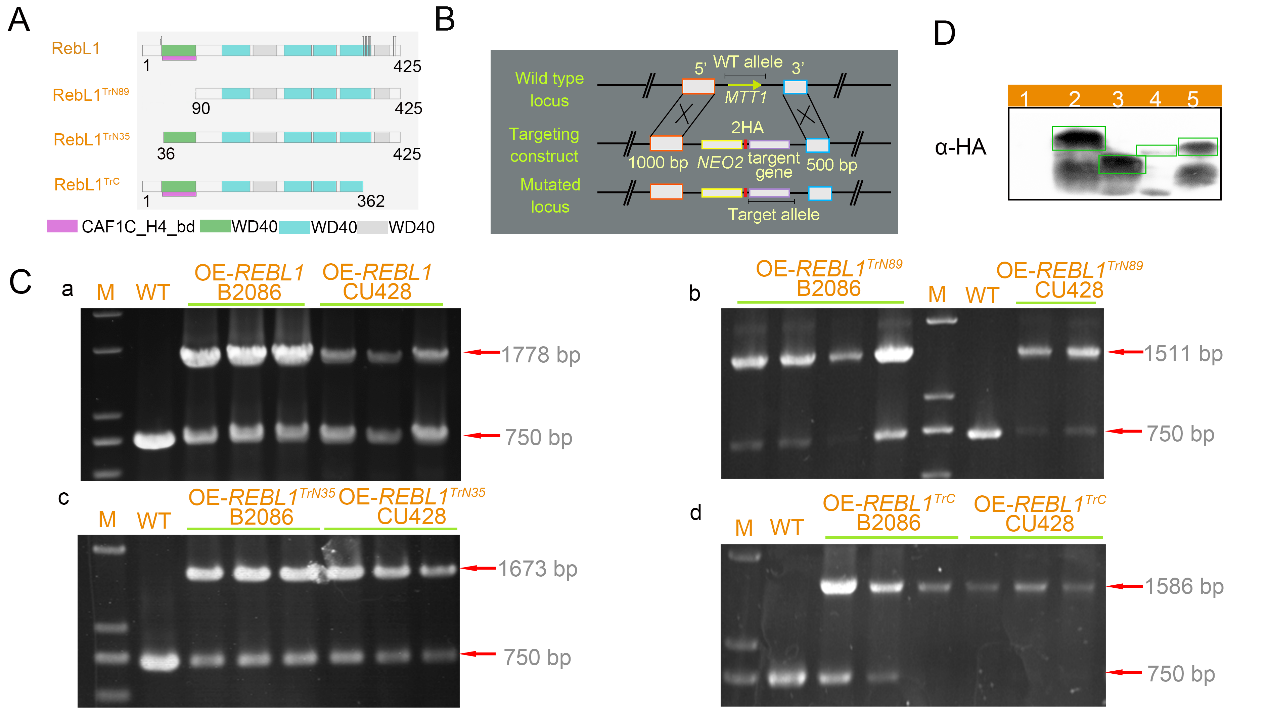


**Fig. S2** Construction and identification of OE-*REBL1*. **A** Schematic of truncated RebL1. Pink rectangles indicate CAF1C_H4_bd domains; green, blue, and gray rectangles mark different types of WD40 repeats. **B** Schematic of the construction of overexpression plasmids. The flanking sequences of *MTT1* denoted as 5' (orange rectangular box) and 3' (blue rectangular box), respectively, represent the regions upstream and downstream of *MTT1*. *NEO2* (yellow rectangular box) refers to the element containing paromomycin resistance, while 2HA (red rectangle) indicates the presence of two tandem HA tags. Purple rectangle refers to target gene. **C** Homologous recombination substitution of strains. Arrows indicate mutated loci and WT loci. **D** Western blot of RebL1 and its truncated protein. Green boxes indicate target proteins; 1–5, WT, HA-RebL1, HA-RebL1^TrN89^ HA-RebL1^TrN35^, and HA-RebL1^TrC^, respectively.


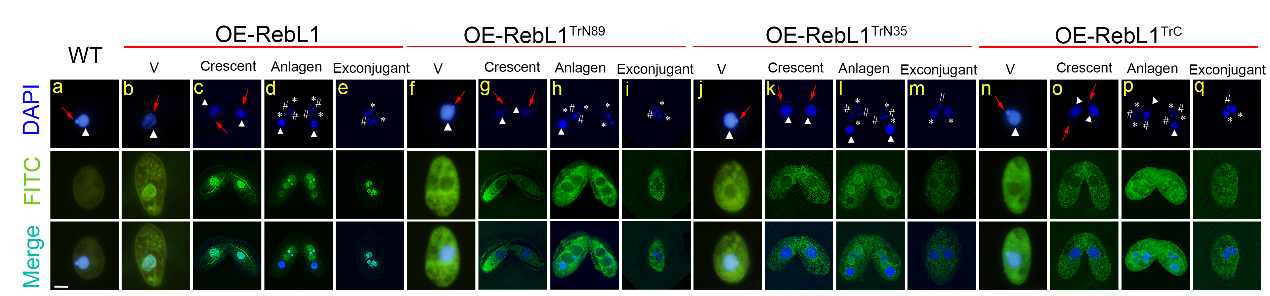


**Fig. S**3 Localization of RebL1 mutants during vegetative growth and conjugation. a, WT; b–e, overexpression of RebL1; f–i, overexpression of RebL1^TrN89^; j–m, overexpression of RebL1^TrN35^; n–q, overexpression of RebL1^TrC^; a, b, f, j and n, vegetative growth; c, g, k and o, crescent; d, h, l and p, anlagen; e, i, m and q, exconjugant with two MACs and one MIC. Triangles indicate MACs, arrows indicate gametic nucleus, # signs indicate zygotic nucleus, and * indicates new MACs (n=20 per period). Scale bar, 10 µm.


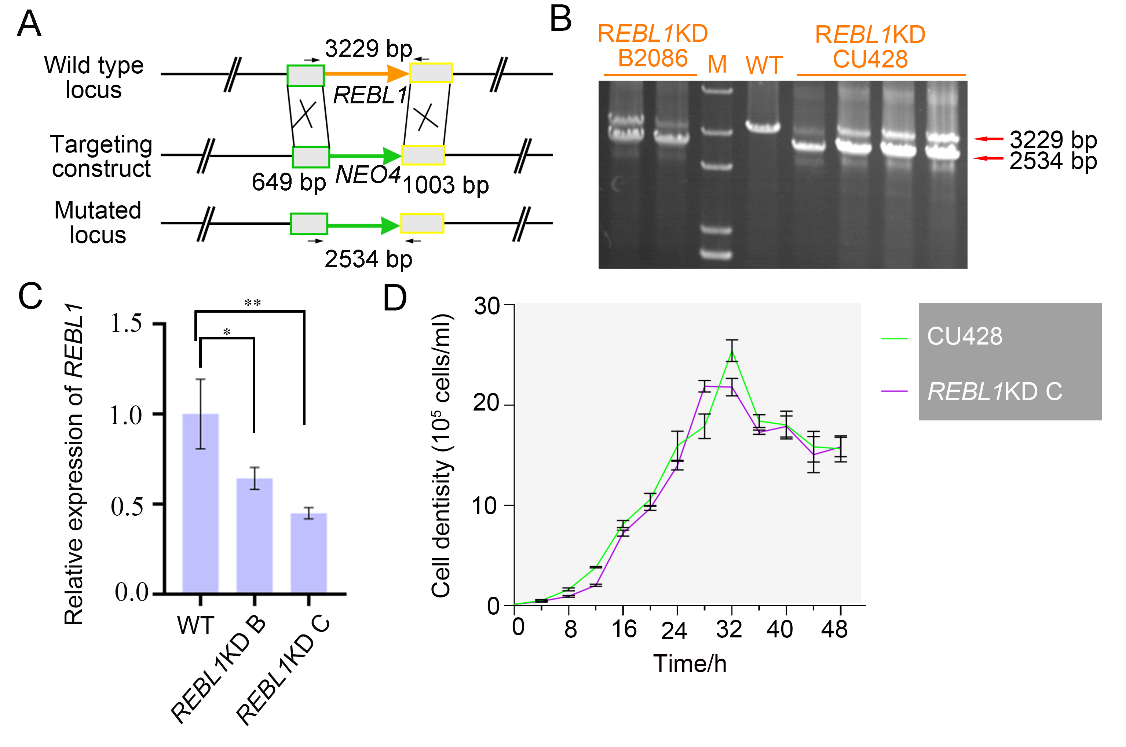


**Fig. S**4 Construction and identification of *REBL1* knockdown mutants. **A** Schematic of *REBL1* knockout. The rectangle in the green box represents the 5' homologous arm of *REBL1*, and the yellow rectangle represents the 3' homologous arm. Yellow arrows indicate *REBL1*, green arrows indicate *NEO4* and black arrows indicate primers used to identify homologous recombination. **B** Homologous recombination substitution of *REBL1*. Arrows indicate mutated loci (2534 bp) and WT loci (3229 bp). **C** Relative expression of *REBL1* in WT and mutants. T-test was applied for significance analysis (**p*<0.05; ***p*<0.01). **D** Growth curve of wild type and mutants in SPP medium. The initial cell concentration is 0.125×10^5^ cells/mL.


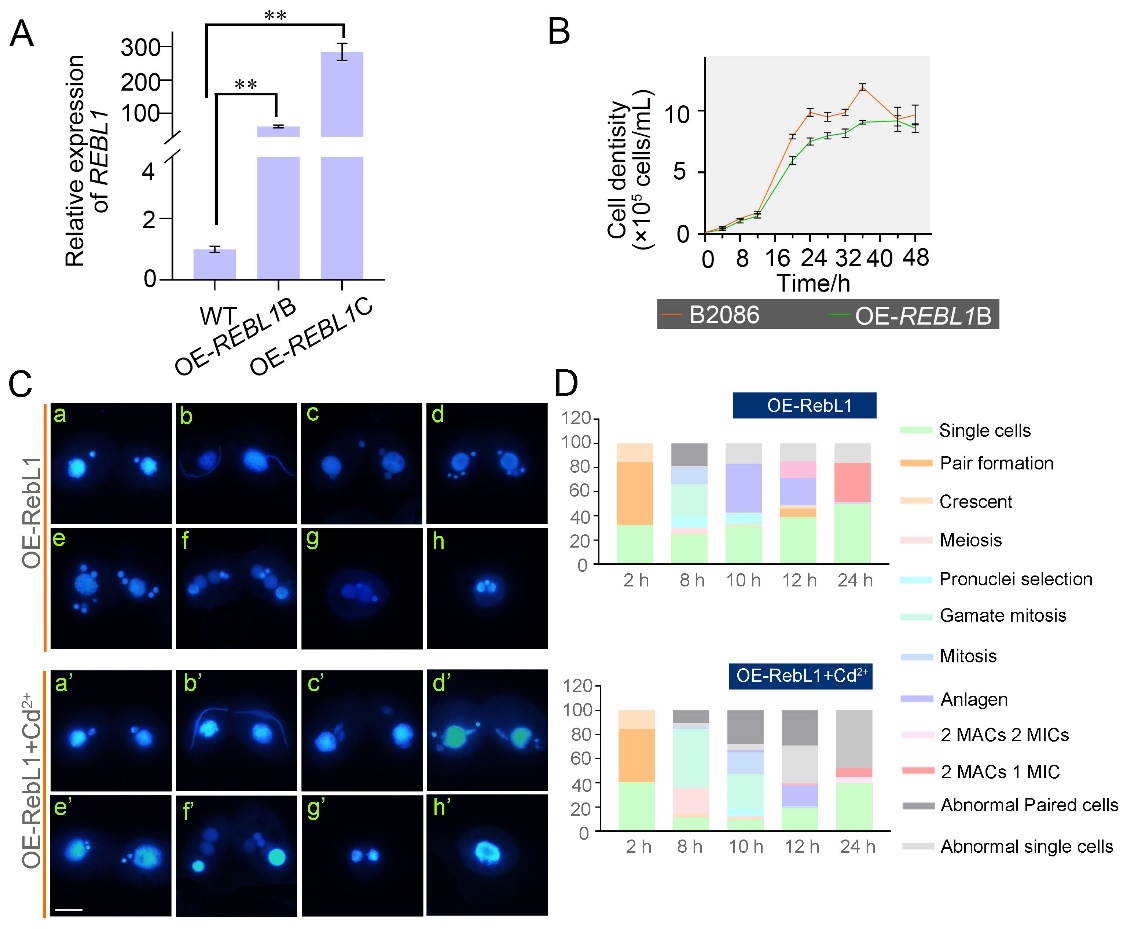


**Fig. S**5 Overexpression of *REBL1* affected mitosis of zygotic nucleus. **A** Relative expression of *REBL1* in OE-*REBL*1B and OE-*REBL*1C mutants. T-test was applied for significance analysis (***p*<0.01). **B** Proliferation of B2086 and OE-RebL1B. The cells were cultured in SPP medium containing 0.5 μg/mL Cd^2+^ with an initial concentration of 0.125×10^5^ cells/mL. **C** Sexual reproductive development of OE-RebL1. a and a’, pair formation; b and b’, crescent; c and c’, meiosis; d and d’, pronuclei selection; e, mitosis; f, anlagen; g, exconjugant with two MACs two MICs; h, exconjugant with two MACs one MIC; e’, abnormal mitosis; f’, abnormal anlagen; g’ and h’, abnormal single cells. Scale bar, 10 µm. **D** Statistics of nuclear development of OE-RebL1 mutant during sexual reproduction (n=300).

**Table S1** RBBP4/RBBP7 function in *Tetrahymena thermophila* and mammals

| RBBP4/RBBP7-containing complex | *T. thermophila* | mammal |
| --- | --- | --- |
| Sin3 (histone deacetylation) | **+** | **+** |
| MuvB (transcription regulation) | **+** | **+** |
| CAF-1 (chromatin assembly) | **+** | **+** |
| HAT (histone acetylation) | **+** | **+** |
| NuRD (Nucleosome remodeling, histone deacetylation) | **+** | **+** |
| NURF (Nucleosome remodeling) | **-** | **+** |
| PRC2 (Methylation of H3K27 and gene regulation) | **-** | **+** |

| **Table S2** Primers used in this article. | |
| --- | --- |
| Primer name | Sequences |
| *REBL1*-HA-5F | GAGCTCCCTCTCCATGATATCGAATTTC |
| *REBL1*-HA-5R | GCGGCCGC GTCTTTCATTACTTCATCATCAGT |
| *REBL1*-HA-3F | CTCGAGTACTTAACTTTCAGCTCTCTGAT |
| *REBL1*-HA-3R | GGTACCTCAATGTACAGCTGGTGTTTC |
| Shoot-*REBL1*-HA-F | CTCTCCATGATATCGAATTTCATAAATC |
| Shoot-*REBL1*-HA-R | GTACCTCAATGTACAGCTGGTGTTT |
| J-*REBL1*-HA-F | CTAGTTGTTCTGCTGATAGAAGAG |
| J-*REBL1*-HA-R | GTAAGTATATTCATTCATACATGCAC |
| K*-REBL1*-5F | GAGCTCGAATGTAATTGCTATTATACAACAAC |
| K-*REBL1*-5R | GCGGCCGCGATATATAAATGATTATTCCGCCTAG |
| K-*REBL1*-3F | CTCGAGGTTCTATTCTTTATCTCCACAACAC |
| K-*REBL1*-3R | GGTACCGATTAGAGGTTGAGATTAAACCTTC |
| J-K-*REBL1*-F | GATGTTAGTCTATTTGTATATGTAG |
| J-K-*REBL1*-R | GCTGAAAGTTAAGTATTTTCGCG |
| RNAi-*REBL1*-1F | CTGCAGACTCATTGTACATAAGCTCATACT |
| RNAi-*REBL1*-1R | CCCGGGCATTTACTTTTGCTCTGTGGCC |
| RNAi*REBL1*-2F | GGATCCACTCATTGTACATAAGCTCATACT |
| RNAi-*REBL1*-2R | GTTTAAACCATTTACTTTTGCTCTGTGGCC |
| RT-*REBL1*-F | GCTACCTACTAAAGATATCCCTTAAG |
| RT-*REBL1*-R | GGCAATCTAACCTTGGCAATAAGC |
| RT-*SIN3*-F | TTCAAATGCAGCTTCTGCAGCTGC |
| RT-*SIN3*-R | GTCATTCTCCTTACGAATCTTGTTG |
| RT-*THD1*-F | GTATCCAGATTAGCCATCTGACAAA |
| RT-*THD1*-R | CGATGTATTCGTCAGAGTGGAAG |
| RT-*CHD3*-F | GATGGGTACTCGTAGAAGATAGTA |
| RT-*CHD3*-R | GGCTTACGCACTGCCTCATCATC |
| RT-*HAT1*-F | CCTCTCTTAGTTTGAGGGTCCAG |
| RT-*HAT1*-R | TTAGCATCTCCGTCTTCTCCTAAG |
| RT-*CAF1B*-F | GGTTATATCAAGTTGTGGTAGATTAAC |
| RT-*CAF1B*-R | CCCTTATCAACATGCCAAATGACAAT |
| RT-*FORC1*-F | GTGTATACAGAAGCTAATCTAGACC |
| RT-*FORC1*-R | TAATTCCTTTATCCTTTGCTACTAGA |
| RT-*POLD1*-F | AGATATTGCCTTGCTTTAAGCTAAC |
| RT-*POLD1*-R | ATCATCTCCTTGATCTCCATCTTTC |
| RT-*RPB1*-F | GAGAGCCCCCTATTAAGAGAAATG |
| RT-*RPB1*-R | TCAAATAAATATGAGTTGCACTTATCG |
| RT-*HIR1*-F | CTTCAAAAAGAGATGACAGAAAAATAGA |
| RT-*HIR1*-R | ATAGATGAAAAAGGCCCCATAAATGTT |
| 17S-F | GATCCTGCCAGTTACATATGCTTG |
| 17S-R | GCCCAACAATTAGCTCGGTTATCC |
| OE-*REBL1*-F | GGATCCATGAATCAATAAATGGCTGAAGAATT |
| OE-*REBL1*-R | GGCGCGCCTCAGTCTTTCATTACTTCATCATCAG |
| OE-*REBL1-*TrN35-F | GGATCCGCAATAACACACGAATTAGAGTGG |
| OE-*REBL1*-TrN35-R | GGCGCGCCTCAGTCTTTCATTACTTCATCATCAG |
| OE-*REBL1-*TrN89-F | GGATCCCTTGAAGAAACTGCTACAGATATTAG |
| OE-*REBL1*-TrN89-R | GGCGCGCCTCAGTCTTTCATTACTTCATCATCAG |
| OE-*REBL1-*TrC-F | GGATCCATGAATCAATAAATGGCTGAAGAATT |
| OE-*REBL1*-TrC-R | GGCGCGCCATTTTTCATTTCCTAACCACATTTG |
| OE-J-*REBL1*-F | GCTACGTGATTCACGATTTATGCAATG |
| OE-J-*REBL1*-R | CGAAACTGATTTTATGCAATTATGAATTAC |
