# Supplementary figures and images for: RebL1 is required for macronuclear structure stability and gametogenesis in Tetrahymena thermophila
Source: Mar Life Sci Technol. 2024 Mar 26;6(2):183–97. doi: 10.1007/s42995-024-00219-z (PMC11136921; doi:10.1007/s42995-024-00219-z)

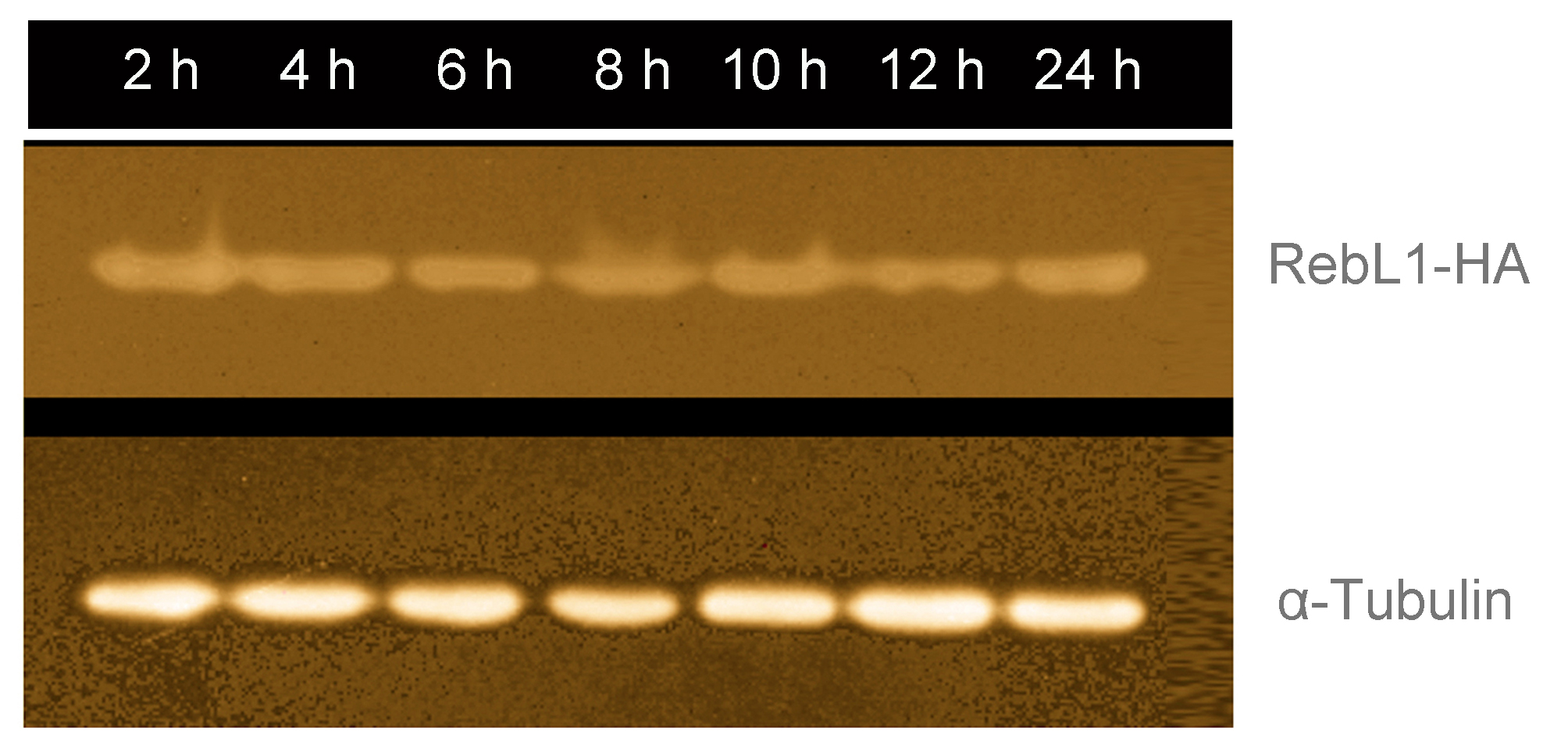

Supplement: Supplementary file 1 — Supplementary file1 (ZIP 8227 KB) [file 42995_2024_219_MOESM1_ESM.zip › 42995_2024_219_MOESM1_ESM/FigS1(8cm) .jpg]

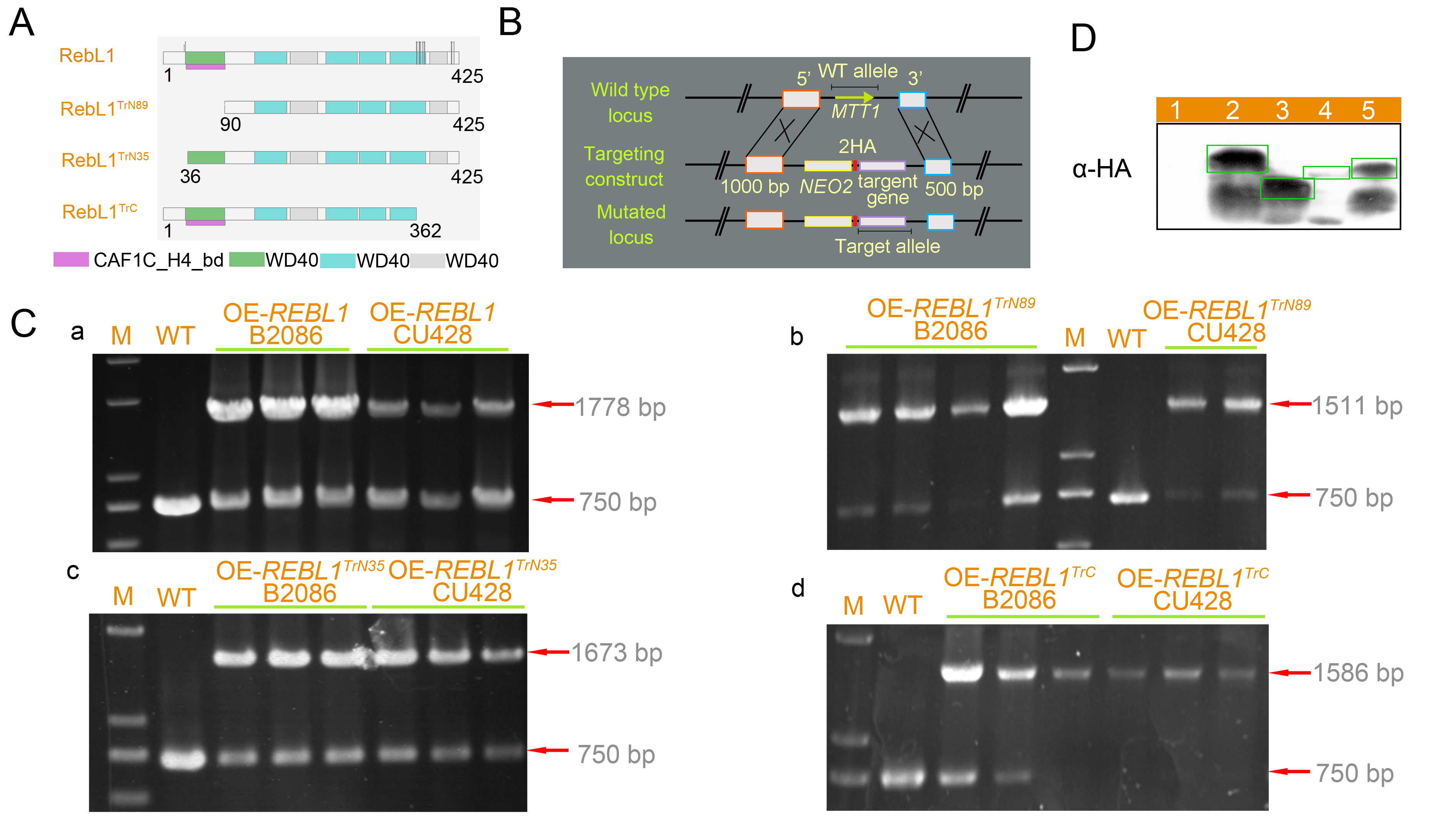

Supplement: Supplementary file 1 — Supplementary file1 (ZIP 8227 KB) [file 42995_2024_219_MOESM1_ESM.zip › 42995_2024_219_MOESM1_ESM/FigS2(16cm).jpg]

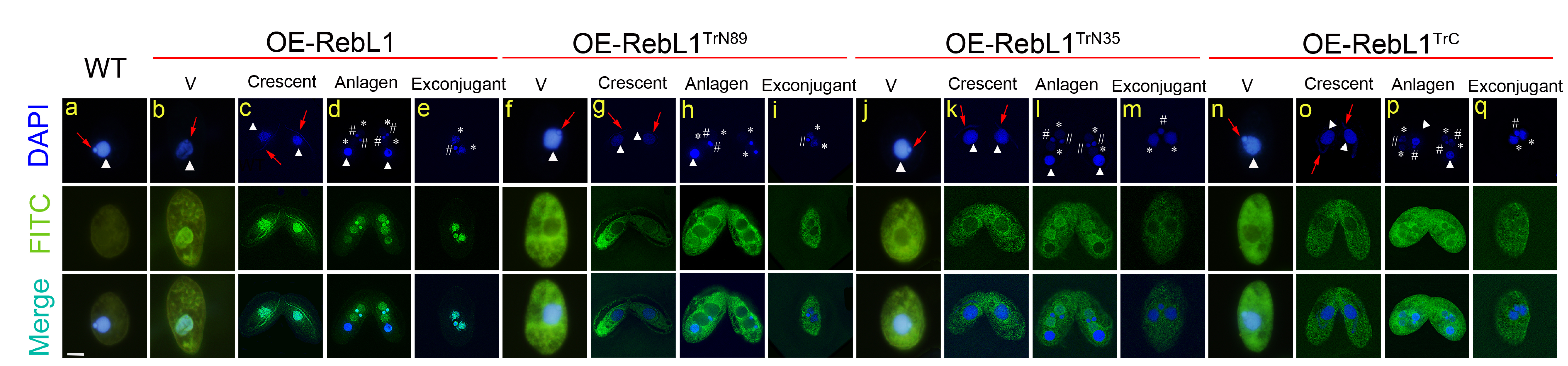

Supplement: Supplementary file 1 — Supplementary file1 (ZIP 8227 KB) [file 42995_2024_219_MOESM1_ESM.zip › 42995_2024_219_MOESM1_ESM/FigS3(16 cm).jpg]

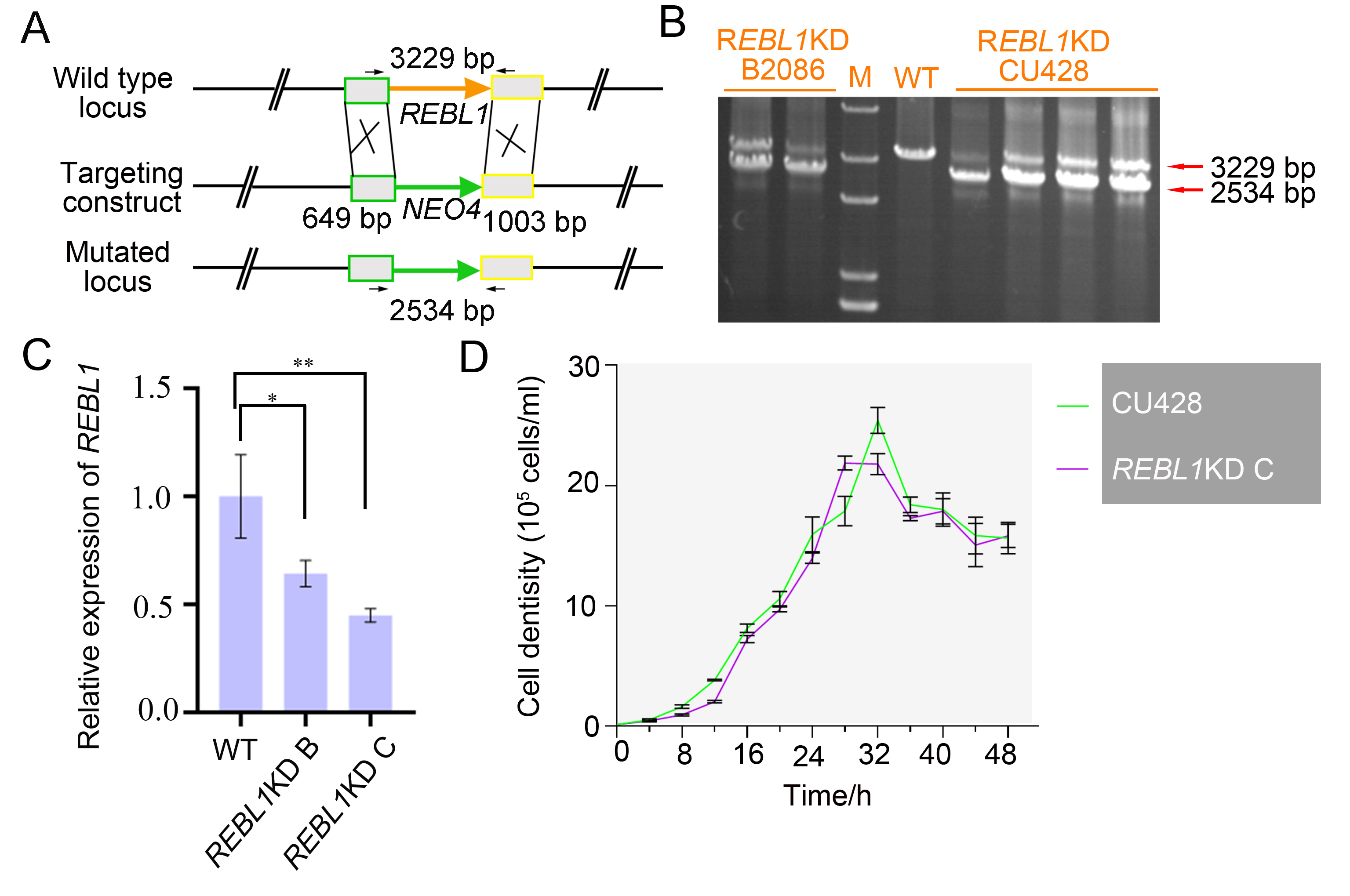

Supplement: Supplementary file 1 — Supplementary file1 (ZIP 8227 KB) [file 42995_2024_219_MOESM1_ESM.zip › 42995_2024_219_MOESM1_ESM/FigS4(13 cm).jpg]

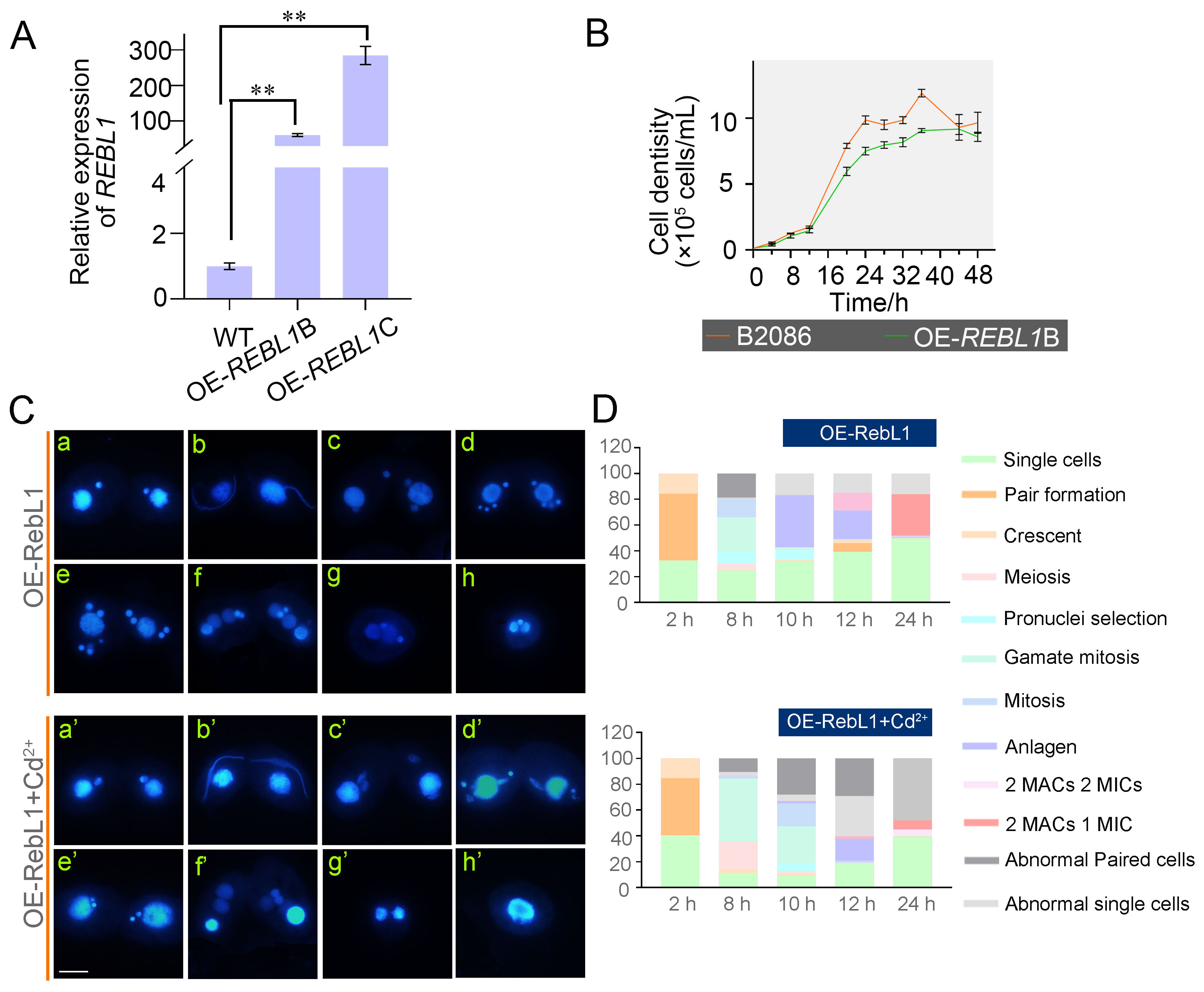

Supplement: Supplementary file 1 — Supplementary file1 (ZIP 8227 KB) [file 42995_2024_219_MOESM1_ESM.zip › 42995_2024_219_MOESM1_ESM/FigS5(16cm).jpg]
